# A CD14‐Targeting In Situ Hydrogel Directs Macrophage Reprogramming to Minimize Scar Formation

**DOI:** 10.1002/advs.77602

**Published:** 2026-09-06

**Authors:** Rui Fang, Simeng Chen, Qiaozhen Liu, Wenwen Ni, Xi Xu, Jianfa Zhang

**Affiliations:** ^1^ Center for Molecular Metabolism Nanjing University of Science & Technology Nanjing People's Republic of China; ^2^ Key Laboratory of Metabolic Engineering and Biosynthesis Technology Ministry of Industry and Information Technology Nanjing People's Republic of China

**Keywords:** CD14, hemoadhican, immune microenvironment, macrophage reprogramming, minimal‐scarring wound repair

## Abstract

Despite the urgent need for therapies that enable high‐quality skin regeneration with minimal scar formation, current wound dressings remain largely passive and provide limited control over the inflammatory and fibrotic processes that determine repair outcomes. Here, a microbial polysaccharide‐based in situ self‐gelling powder, Hemoadhican (HD), is shown to actively reprogram the wound microenvironment by targeting macrophage CD14. Upon contact with wound exudate or blood, HD rapidly forms a stable, tissue‐adhesive hydrogel with excellent biocompatibility. In mouse deep second‐degree burn, rabbit ear hypertrophic scar, and rat abdominal incision models, HD significantly accelerates wound closure, promotes organized collagen remodeling, reduces scar formation, and enhances the regeneration of hair follicles, blood vessels, and neural structures. Protein affinity‐capture assays identify CD14 as a specific binding receptor for HD. Mechanistically, CD14 engagement by HD reprograms macrophage responses, suppressing nuclear factor‐κB (NF‐κB) signaling and altering the macrophage‐derived paracrine microenvironment, which subsequently enhances endothelial protein kinase B (AKT) phosphorylation and reduces fibroblast SMAD family member 2 (Smad2) phosphorylation to coordinate angiogenesis and fibrotic remodeling. These findings reveal a previously unrecognized CD14‐targeting function of a microbial polysaccharide and establish an in situ self‐gelling immunomodulatory platform that actively reprograms the wound microenvironment to enable high‐quality skin regeneration.

## Graphical abstract

A CD14‐targeting in situ hydrogel formed from a microbial polysaccharide rapidly remodels the immune microenvironment by reprogramming macrophage polarization, suppressing excessive inflammation, and coordinating tissue regeneration. This multifunctional hydrogel promotes scar‐reduced wound repair through enhanced angiogenesis, collagen remodeling, and neural reconstruction, providing a promising strategy for high‐quality tissue regeneration.

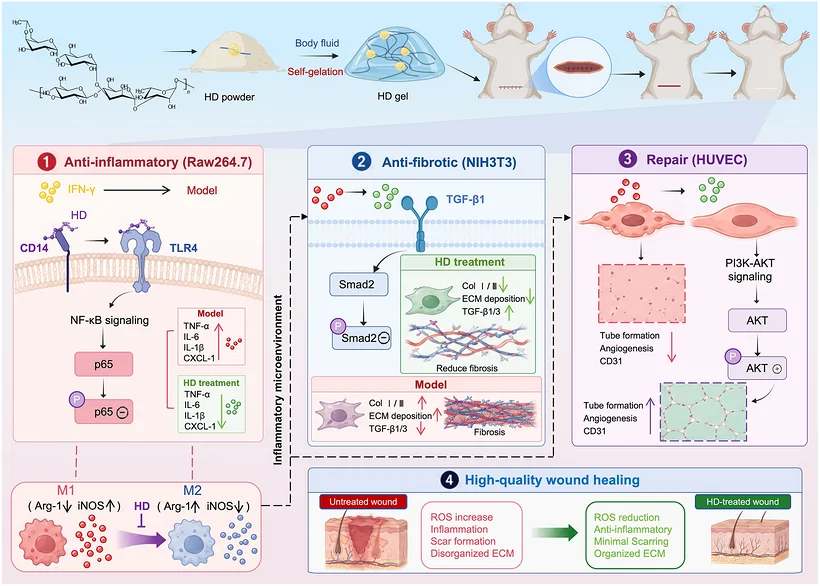

## 1 Introduction

As the body's largest organ and primary immune defense barrier, the skin plays a crucial role in maintaining internal homeostasis by preventing excessive water loss, regulating body temperature, sensing external stimuli, and defending against pathogenic microorganisms [1, 2, 3, 4]. When skin integrity is compromised, the body initiates a complex wound‐healing process to restore the tissue's barrier function [5, 6]. However, in adult mammals, this process typically results in fibrosis‐dominated repair, ultimately leading to scar formation [7]. Deep skin injuries (such as trauma, burns, and surgical wounds) often result in excessive scarring, which not only compromises tissue structure and functional integrity but also causes significant psychological distress and cosmetic impairment [8]. In developed countries, more than 100 million people are affected by scar‐related issues. This prevalence is largely due to current treatment strategies struggling to effectively reconstruct the normal extracellular matrix (ECM) architecture, restore the regenerative capacity of skin appendages, and remodel the tissue's mechanical properties [9, 10, 11].

A growing body of research indicates that pathological scar formation is not merely a byproduct of tissue repair but rather an abnormal outcome driven by an imbalanced wound microenvironment. Specifically, persistent inflammatory responses, dysregulated oxidative stress, and disrupted ECM remodeling collectively constitute key factors that impede tissue regeneration [12, 13]. Following injury, sustained activation of the innate immune system leads to prolonged release of pro‐inflammatory factors, which further induce abnormal fibroblast activation and excessive collagen deposition, thereby driving the fibrotic process [14, 15]. Concurrently, persistent oxidative stress exacerbates tissue damage and disrupts the homeostasis of the regenerative microenvironment, ultimately causing wound healing to deviate from the regenerative pathway and enter an irreversible scarring process [16]. Currently, commonly used clinical scar management strategies, including silicone therapy, laser intervention, and compression therapy, primarily focus on the scar remodeling phase after epithelialization has been completed rather than preventing scar formation at its source [17, 18]. Although these methods improve scar appearance to some extent, their efficacy remains limited, treatment durations are relatively long, and costs are high [19]. More importantly, these strategies do not directly intervene in the early biological processes that determine the outcome of tissue repair. Therefore, proactively regulating immune responses and tissue remodeling during the early stages of wound healing to promote the transition from fibrotic repair to regenerative repair remains a key unresolved scientific question in the field of regenerative medicine [20].

Traditional wound dressings, such as gauze and polymer films, primarily function by providing physical isolation to reduce external contamination and lower the risk of infection. However, they have limited efficacy in promoting tissue regeneration and regulating key pathological processes, including infection, inflammation, and immune imbalance [21]. Moreover, these materials often fail to effectively inhibit abnormal collagen deposition, which can lead to hypertrophic scar formation, thereby compromising skin elasticity and adversely affecting the cosmetic outcome of wound healing [22]. Consequently, there is an urgent need for advanced wound care strategies that not only protect the wound but also actively modulate the wound microenvironment to promote faster and higher‐quality healing. Achieving this goal largely depends on regulating inflammatory responses, maintaining reactive oxygen species (ROS) homeostasis, and restoring normal ECM remodeling [11, 23]. The excellent biocompatibility of polysaccharides, along with their immunomodulatory and antibacterial properties, has garnered widespread attention in the field of wound repair. Common polysaccharides used in wound care include cellulose, hemicellulose, starch, alginate, dextran, and hyaluronic acid [24, 25]. However, there remains a lack of clear and consistent mechanistic understanding regarding whether polysaccharide‐based materials can systematically regulate inflammatory responses, oxidative stress, and dynamic ECM remodeling in complex or deep wounds, and whether they can further influence repair outcomes and scar formation.

Hemoadhican (HD) is a naturally occurring polysaccharide composed of hexasaccharide repeating units [26]. Our group has previously demonstrated that HD exhibits potent hemostatic activity independent of the coagulation cascade [27]. Additionally, HD‐based materials effectively reduce inflammation, mitigate abnormal collagen deposition, and prevent tissue adhesions in various preclinical models [28, 29]. These findings prompted us to investigate whether HD can actively modulate the wound microenvironment beyond providing passive physical protection. Here, we developed an in situ self‐gelling HD powder dressing and systematically evaluated its physicochemical properties, biocompatibility, and therapeutic efficacy across multiple wound models, including deep second‐degree burns, rabbit ear hypertrophic scars, and abdominal incisions under high tension. Furthermore, receptor screening combined with molecular and cellular analyses was conducted to elucidate the pro‐regenerative mechanism of HD. This study aims to establish a naturally derived, in situ gelling dressing capable of promoting high‐quality wound repair while providing mechanistic insights into its immunoregulatory mode of action.

## 2 Results

### 2.1 A Blood‐Triggered In Situ Gelling Powder With Excellent Tissue Adhesion and Biosafety

**FIGURE 1:**
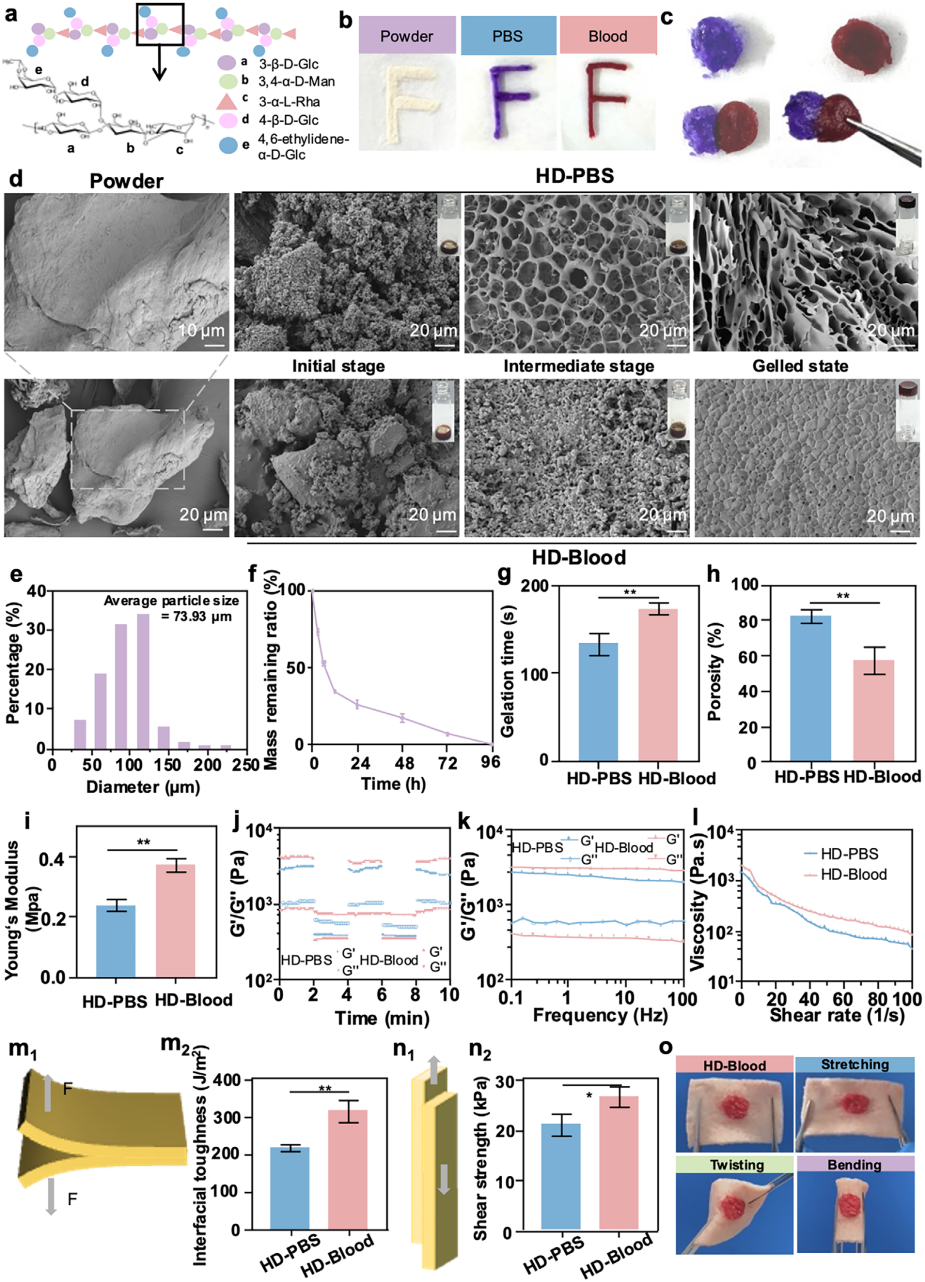
Characterization of the in situ self‐gelling behavior and adhesive properties of HD. (a) Predicted Haworth structure of HD. (b) Representative photographs of HD powder, HD‐PBS gel, and HD‐Blood gel. (c) Self‐healing behavior of HD‐PBS gel and HD‐Blood gel after mechanical disruption. (d) Scanning electron microscopy (SEM) images of HD powder, HD‐PBS gel, and HD‐Blood gel. (e) Particle size and (f) in vitro degradation of HD. (g) Gelation time, (h) porosity, and (i) Young's Modulus of HD‐PBS gel and HD‐Blood gel (*n* = 3). (j) Cyclic strain sweep rheological analysis. (k) Storage modulus (G′) and loss modulus (G″) as functions of frequency (0.1–100 Hz). (l) Apparent viscosity as a function of shear rate (0.01–100 s^−^^1^). (m_1_) Schematic illustration of the 180° peel test. (m_2_) Quantification of interfacial fracture toughness (*n* = 3). (n_1_) Schematic illustration of the lap‐shear test. (n_2_) Quantification of lap‐shear strength (*n* = 3). (o) Representative photographs showing the adhesion of HD‐Blood gel to porcine skin under different mechanical deformations. Data are presented as mean ± SD. Statistical significance was determined using Student's t‐test (^*^ *p* < 0.05, ^**^ *p* < 0.01). Error bars represent standard deviation.

To address the unmet need for dressings that actively promote minimal‐scarring wound healing, we developed an in situ self‐gelling powder based on the microbial polysaccharide HD. Prepared via lyophilization and grinding (Figure 1a), HD rapidly swelled and self‐assembled into a stable hydrogel upon contact with liquid (Figure 1b). The gel formed in blood appeared red (HD‐Blood), whereas the gel formed in phosphate‐buffered saline (PBS) (HD‐PBS) was stained purple for easier visualization. Both systems exhibited remarkable self‐healing behavior, readily re‐fusing after shear‐induced rupture, indicating dynamic network reconstruction and structural stability in both aqueous and blood environments (Figure 1c). Scanning electron microscopy (SEM) revealed distinct structural transformations of HD under different conditions (Figure 1d). HD powder displayed a dense block‐like morphology with a smooth surface and few pores. After hydration with PBS, HD rapidly formed a three‐dimensional honeycomb‐like porous network with uniform pores and high connectivity. Upon blood exposure, the HD–Blood system underwent progressive restructuring, characterized by particle aggregation, interparticle bridging, and formation of a dense microporous gel network, indicating rapid integration with blood components. HD showed an average particle size of 73.93 µm, favorable for rapid hydration and homogeneous gel formation (Figure 1e). In PBS, HD underwent gradual degradation and was completely degraded within 96 h, suggesting a balanced degradation profile that preserves temporary integrity while allowing tissue regeneration (Figure 1f) [30]. Compared with HD‐PBS gel, HD‐Blood gel exhibited lower porosity (57.47% ± 7.58% vs. 82.28% ± 3.88%) and slightly prolonged gelation time (Figure 1g,h), reflecting the incorporation of blood cells and plasma proteins into the network, which enhanced gel compactness and stability.

The mechanical properties of HD gels were further evaluated by Young's modulus analysis (Figure 1i). Both PBS‐ and blood‐induced HD gels exhibited stable mechanical properties, while HD‐Blood showed a significantly higher Young's modulus (0.370 ± 0.024 MPa) than HD‐PBS (0.238 ± 0.020 MPa), demonstrating that blood components reinforced the gel network. Previous studies have shown that hydrogel stiffness regulates wound repair by affecting wound contraction, keratinocyte proliferation, migration, and extracellular matrix remodeling, thereby influencing scar formation [31]. Thus, the moderate stiffness of HD‐Blood gel may provide a favorable biomechanical microenvironment for tissue repair, synergizing with its immunomodulatory effects. Rheological analysis further characterized the viscoelastic properties of the HD gels. Alternating step‐strain tests (Figure 1j) demonstrated that under low‐strain conditions, the storage modulus (G′) of both the HD‐PBS and HD‐Blood gels was significantly higher than the loss modulus (G″). Under high‐strain conditions, both systems underwent a reversible gel–sol transition, indicating excellent structural recoverability and self‐healing capability. Notably, the HD‐Blood gel exhibited a substantially higher modulus than the HD‐PBS gel, suggesting that blood components enhanced network cross‐linking and mechanical strength. Frequency sweep measurements (0.01–100 Hz, 1% strain) further confirmed stable viscoelastic behavior, with G′ consistently exceeding G″ throughout the tested frequency range. Moreover, the HD‐Blood gel maintained higher G′ values than the HD‐PBS gel across the entire frequency range (Figure 1k). Shear‐rate sweep analysis also showed that the HD‐Blood gel exhibited a higher apparent viscosity than the HD‐PBS gel (Figure 1l).

As the human body is constantly in motion, wound dressings are frequently subjected to deformation, which can compromise their adhesive performance. Additionally, wound exudate and blood create a moist environment that generally weakens tissue adhesion. Therefore, maintaining strong adhesion under both dynamic and wet conditions is essential [32]. We further evaluated the tissue adhesive properties of the HD gels. The 180° peel test demonstrated that the HD‐Blood gel exhibited significantly stronger adhesion to wet porcine skin than the HD‐PBS gel (Figure 1m_1_), with an interfacial fracture toughness of 312.85 ± 29.39 J/m^2^ (Figure 1m_2_). This enhanced adhesion is primarily attributed to the synergistic effects of extensive hydrogen bonding and topological entanglement arising from polymer chain diffusion [26]. Consistently, lap shear testing showed that the HD‐Blood gel exhibited a significantly higher interfacial shear strength (26.24 ± 1.96 kPa) than the HD‐PBS gel (20.86 ± 2.27 kPa) (Figure 1n_1–2_). Furthermore, the in situ‐formed HD‐Blood gel maintained stable adhesion under mechanical deformation, including stretching, bending, and twisting, demonstrating excellent adaptability to dynamic tissue interfaces (Figure 1o).

Biosafety assessment revealed no significant cytotoxicity at any tested concentration (Figure S1) and a hemolysis rate of approximately 1.14%, well below the 5% safety threshold (Figure S2). Collectively, these results establish HD as a facile, robust, and biocompatible in situ gelling system with excellent tissue adhesion, providing a strong foundation for its evaluation in wound healing and immune microenvironment modulation. In vivo, FITC‐labeled HD fluorescence at the implantation site declined by 72–96 h, with transient signals in plasma and major organs indicating systemic clearance (Figure S3). Histological analysis revealed progressive host cell infiltration from the periphery to the interior as degradation proceeded (Figure S4). This degradation–infiltration timeline paralleled wound healing: HD maintained structural integrity during early inflammation (24–48 h) and degraded to create space for cell migration and remodeling in the proliferative.

### 2.2 Accelerated Wound Closure and Epithelialization in Deep Second‐Degree Burns

**FIGURE 2:**
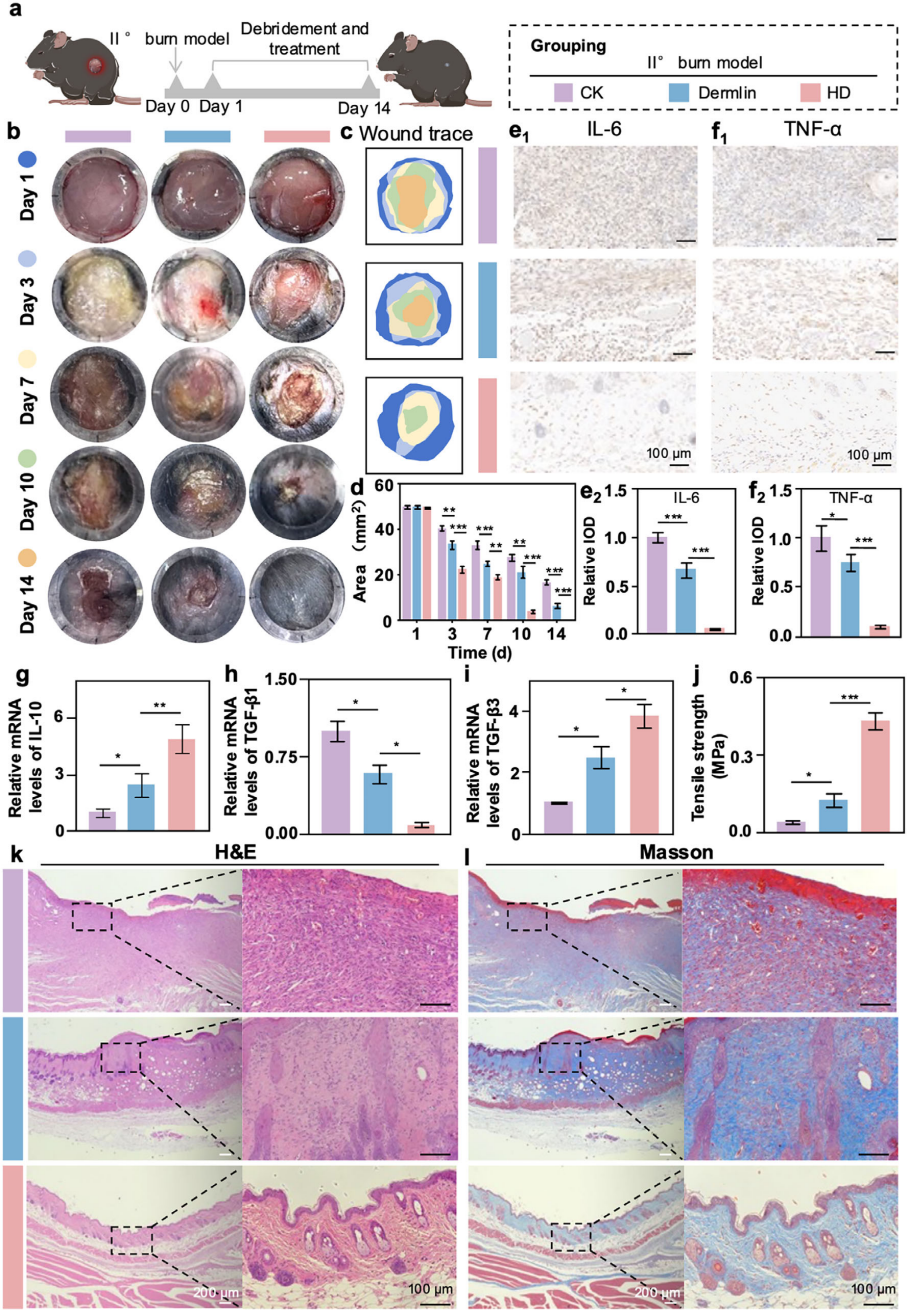
HD accelerates burn wound healing and improves tissue regeneration in vivo. (a) Schematic illustration of the deep second‐degree burn model and treatment protocol. (b) Representative photographs of wounds during the healing process. (c) Representative wound closure traces. (d) Quantification of wound closure (*n* = 6). Representative images and quantitative analyses of immunohistochemical staining for (e_1–2_) IL‐6 and (f_1–2_) TNF‐α on day 7. Relative mRNA expression levels of (g) IL‐10, (h) TGF‐β1, and (i) TGF‐β3 in wound tissues determined by RT‐qPCR (*n* = 3). (j) Tensile strength of healed skin. Representative H&E (k) and Masson's trichrome (l) staining on day 14. Statistical significance was analyzed using one‐way ANOVA (^*^ *p* < 0.05, ^**^ *p* < 0.01, ^***^ *p* < 0.001). Error bars represent standard deviation.

Burn injuries are a common and severe form of skin injury, with deep second‐degree burns often accompanied by persistent inflammation and significant pathological scar formation [33]. To assess the therapeutic potential of HD powder in burn wound repair, a mouse model of deep second‐degree burns was established (Figure 2a), and wound healing was continuously monitored across different treatment groups from days 0 to 14 (Figure 2b,c). The HD‐treated group achieved nearly complete wound closure by day 14, significantly outperforming both the control and Dermlin groups. In contrast, the control group exhibited a larger wound area and markedly delayed healing. Quantitative analysis further confirmed that the wound area in the HD group remained significantly smaller than in the other groups throughout the treatment period (Figure 2d), indicating sustained promotion of wound contraction and closure.

Inflammation‐related factors were evaluated on day 7 after treatment. Immunohistochemical analysis showed that the control group exhibited the highest expression levels of IL‐6 and TNF‐α, followed by the Dermlin group, whereas HD treatment significantly reduced the expression of both cytokines (Figure 2e,f). Reverse transcription quantitative polymerase chain reaction (RT‐qPCR) analysis further demonstrated that HD significantly increased the expression of the anti‐inflammatory cytokine IL‐10 (Figure 2g). Additionally, HD significantly downregulated TGF‐β1 mRNA expression while upregulating TGF‐β3 expression, thereby markedly increasing the TGF‐β3/TGF‐β1 ratio (Figure 2h,i). This suggests that HD could suppress excessive fibrosis following burn injury and reduce the risk of scar formation [34]. To further evaluate the quality of tissue repair, uniaxial tensile testing was performed on the regenerated skin. The HD group exhibited significantly greater tensile strength than the other groups, indicating that HD not only accelerated wound closure but also improved the mechanical integrity and functional recovery of the regenerated skin (Figure 2j).

Key indicators of high‐quality wound repair include wound closure, re‐epithelialization, and the regeneration of skin appendages such as hair follicles and sebaceous glands [35]. Histological analyses further revealed significant differences in tissue repair quality among the treatment groups. Hematoxylin and eosin (H&E) and Masson's trichrome staining demonstrated that the control group exhibited pronounced inflammatory cell infiltration, disrupted epidermal architecture, and incomplete re‐epithelialization. Although the Dermlin group showed partial improvement in inflammation, the regeneration of skin appendages, particularly hair follicles, remained limited. In contrast, the HD group significantly promoted wound closure and epithelial regeneration within 14 days, enhanced the regeneration of hair follicles and glands, and concurrently suppressed abnormal collagen deposition (Figure 2k,l). Overall, HD facilitated coordinated restoration of both tissue structure and function, resulting in repair outcomes that more closely resembled normal skin.

**FIGURE 3:**
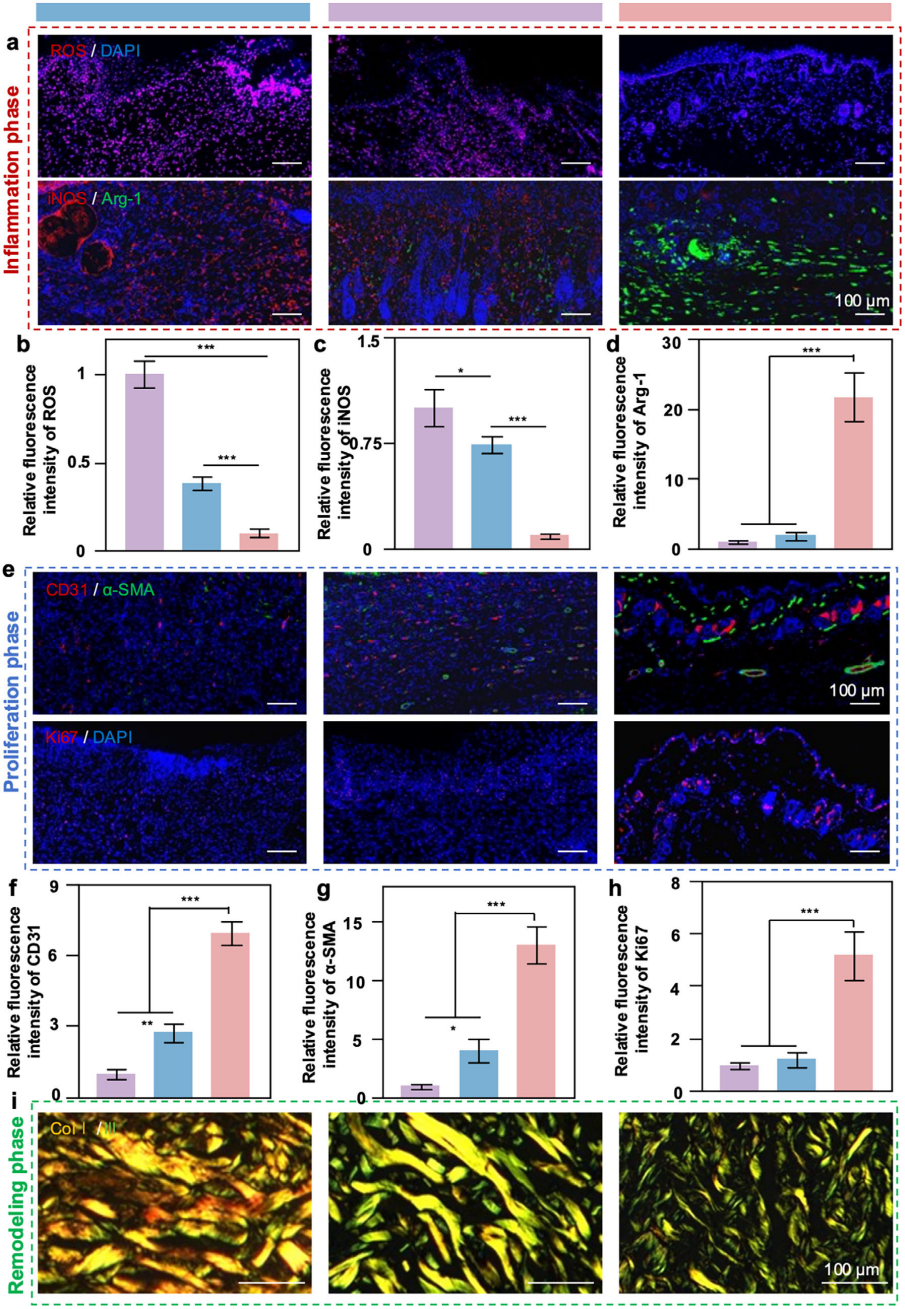
HD attenuates oxidative stress, promotes macrophage polarization, angiogenesis, and collagen remodeling in burn wounds. (a) Representative immunofluorescence images of DCFH‐DA, iNOS, and Arg‐1 staining. Quantification of (b) DCFH‐DA fluorescence intensity, (c) iNOS, and (d) Arg‐1 expression (*n* = 3). (e) Representative immunofluorescence images of CD31, α‐SMA, and Ki67 staining. Quantification of (f) CD31, (g) α‐SMA, and (h) Ki67 expression (*n* = 3). (i) Representative Sirius Red staining of wound tissues (*n* = 3). Statistical significance was analyzed using one‐way ANOVA (^*^ *p* < 0.05, ^**^ *p* < 0.01, ^***^ *p* < 0.001). Error bars represent standard deviation.

Wound healing comprises three distinct yet highly overlapping phases: inflammation, proliferation, and remodeling [36]. We further investigated the regulatory effects of HD during these phases using multiplex immunofluorescence analysis. The inflammatory phase is often prolonged following severe burn injury and is characterized by excessive accumulation of ROS and pro‐inflammatory M1 macrophages. On day 7, 2',7'‐dichlorodihydrofluorescein diacetate (DCFH‐DA) staining and iNOS/Arg‐1 immunofluorescence staining were performed on wound tissues to evaluate the dual functions of HD in ROS scavenging and inflammatory regulation in vivo (Figure 3a). HD treatment markedly reduced DCFH‐DA fluorescence intensity, indicating effective scavenging of intracellular ROS within the wound tissue (Figure 3b). Moreover, compared with the control (CK) group, HD significantly decreased iNOS expression while increasing Arg‐1 expression, confirming macrophage repolarization from the M1 to the M2 phenotype (Figure 3c,d). These findings indicate that HD accelerates the transition from the inflammatory phase to the proliferative phase during burn wound healing.

The proliferative phase is characterized by angiogenesis and cell proliferation, which are essential for supplying oxygen and nutrients to regenerating tissue [37, 38]. Dual immunofluorescence staining was performed using cluster of differentiation 31 (CD31) as an endothelial marker and α‐smooth muscle actin (α‐SMA) as a marker of mature blood vessels to evaluate neovascularization (Figure 3e). After 14 days of treatment, both neovascularization and vascular maturation were significantly enhanced in the HD group compared to the other groups (Figure 3f,g). Additionally, the expression of the proliferation marker Ki67 was significantly increased following HD treatment (Figure 3h). These findings demonstrate that HD effectively promotes angiogenesis and cell proliferation during the proliferative phase of wound healing.

The final stage of wound healing is the remodeling phase. Sirius Red staining was used to analyze collagen deposition and remodeling in the wound. The results demonstrated that HD significantly reduced excessive Type I collagen (COL‐I) deposition and corrected the imbalance in the COL‐I/III ratio, suggesting that it can inhibit scar‐like collagen remodeling and promote an ECM tissue structure more similar to that of normal skin (Figure 3i) [10]. HD effectively suppressed the elevated COL‐I/III ratio in wounds, indicating its strong potential for reduced scar formation (Figure S5). Quantitative analysis of collagen fiber orientation demonstrated distinct differences among the three groups (Figure S6). The CK group exhibited an almost uniform angular distribution, indicating a random collagen organization with no preferred orientation. In contrast, the Dermlin group showed a moderate increase in collagen alignment, with fibers displaying a tendency to orient toward the 0° axis. Notably, the HD group exhibited a markedly narrower angular distribution, with most collagen fibers concentrated between −20° and 20°, indicating a highly ordered and uniformly aligned collagen architecture. These findings confirm that HD facilitates high‐quality, accelerated healing of burn wounds by coordinately regulating the three key stages: inflammatory resolution, angiogenesis, and collagen remodeling.

### 2.3 Suppression of Hypertrophic Scar Formation in a Rabbit Ear Model

**FIGURE 4:**
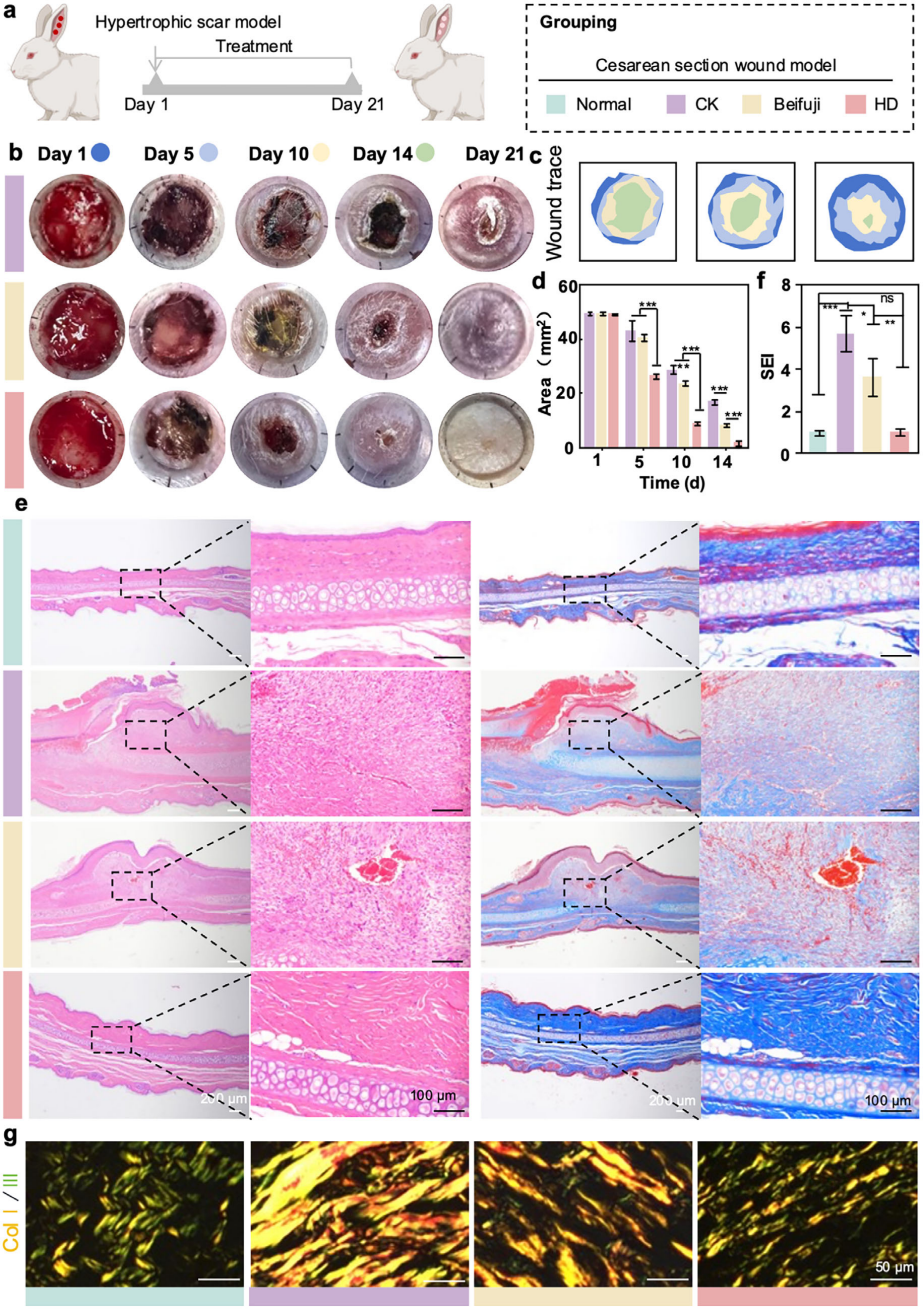
HD suppresses hypertrophic scar formation in the rabbit ear model. (a) Schematic illustration of the rabbit ear hypertrophic scar model and treatment protocol. (b) Representative photographs of wounds during healing. (c) Representative wound closure traces. (d) Quantification of wound closure (*n* = 6). (e) Representative H&E and Masson's trichrome staining on day 21. (f) Quantification of the scar elevation index (SEI) (*n* = 3). (g) Representative Sirius Red staining on day 21. Statistical significance was analyzed using one‐way ANOVA (^*^ *p* < 0.05, ^**^ *p* < 0.01, ^***^ *p* < 0.001). Error bars represent standard deviation.

Because the skin on the dorsum of rodents is highly lax and lacks sustained mechanical tension, wound healing primarily depends on wound contraction, which differs significantly from the skin repair process in humans. In contrast, the rabbit ear wound model lacks a subcutaneous muscle layer, preventing wound closure by contraction. This results in delayed re‐epithelialization and reproducible hypertrophic scar (HS) formation. Consequently, this model is widely regarded as one of the most classic and reliable animal models for studying human HS formation and therapeutic interventions [39] (Figure 4a). Throughout the entire wound healing process, HD not only accelerated wound closure but also significantly inhibited hypertrophic scar formation (Figure 4b–d). Starting on day 5 after injury, wound closure in the HD group was markedly faster, with the residual wound area reduced to 26.29% ± 0.91%, which was significantly smaller than that in the other treatment groups. By day 21, the PBS group still exhibited obvious scab formation and pronounced scar thickening. Although the Beifuji group promoted wound closure, localized scar elevation remained evident. In contrast, wounds treated with HD achieved complete closure and displayed a smooth surface, uniform coloration, and tissue elasticity that more closely resembled normal skin.

The effect of HD on scar remodeling was further evaluated through histological analysis (Figure 4e). H&E staining revealed that the Normal group exhibited intact skin architecture without significant inflammatory infiltration, whereas the CK group showed pronounced epidermal thickening, disorganized dermal structure, and extensive inflammatory cell infiltration. Although inflammation was partially reduced in the Beifuji group, noticeable tissue hyperplasia persisted. In contrast, HD treatment largely restored normal skin architecture, characterized by a well‐organized dermis, markedly decreased inflammatory cell infiltration, and a more uniform distribution of newly formed blood vessels. Masson's trichrome staining further demonstrated extensive collagen overdeposition and disorganized collagen bundles in the CK group, while collagen fibers in the HD group were uniformly distributed and regularly aligned, closely resembling those of normal skin.

To quantitatively assess scar formation, the Scar Elevation Index (SEI) was calculated. HD treatment significantly reduced scar elevation, with an SEI value approaching that of normal tissue (HD group: 1.02 ± 0.18; Normal group: 1.00 ± 0.12), indicating that HD effectively suppressed pathological scar formation (Figure 4f). Furthermore, Sirius Red staining revealed that the control group exhibited densely packed type I collagen fibers arranged in thick bundles, whereas HD treatment resulted in a more uniform and well‐organized collagen architecture with markedly reduced deposition of mature collagen (Figure 4g and Figure S7). Collagen fiber orientation analysis revealed a disorganized distribution in the CK group. Compared with the CK and Beifuji groups, the HD group exhibited a more concentrated collagen fiber orientation, approaching that of normal skin. These results indicate that HD promotes more orderly collagen remodeling during wound healing (Figure S8). Collectively, these findings demonstrate that HD not only accelerates wound healing in the rabbit ear model but also effectively attenuates hypertrophic scar formation by suppressing persistent inflammation and promoting physiological collagen remodeling.

### 2.4 HD Enhances the Early Repair Microenvironment of Abdominal Incisions in Rats

**FIGURE 5:**
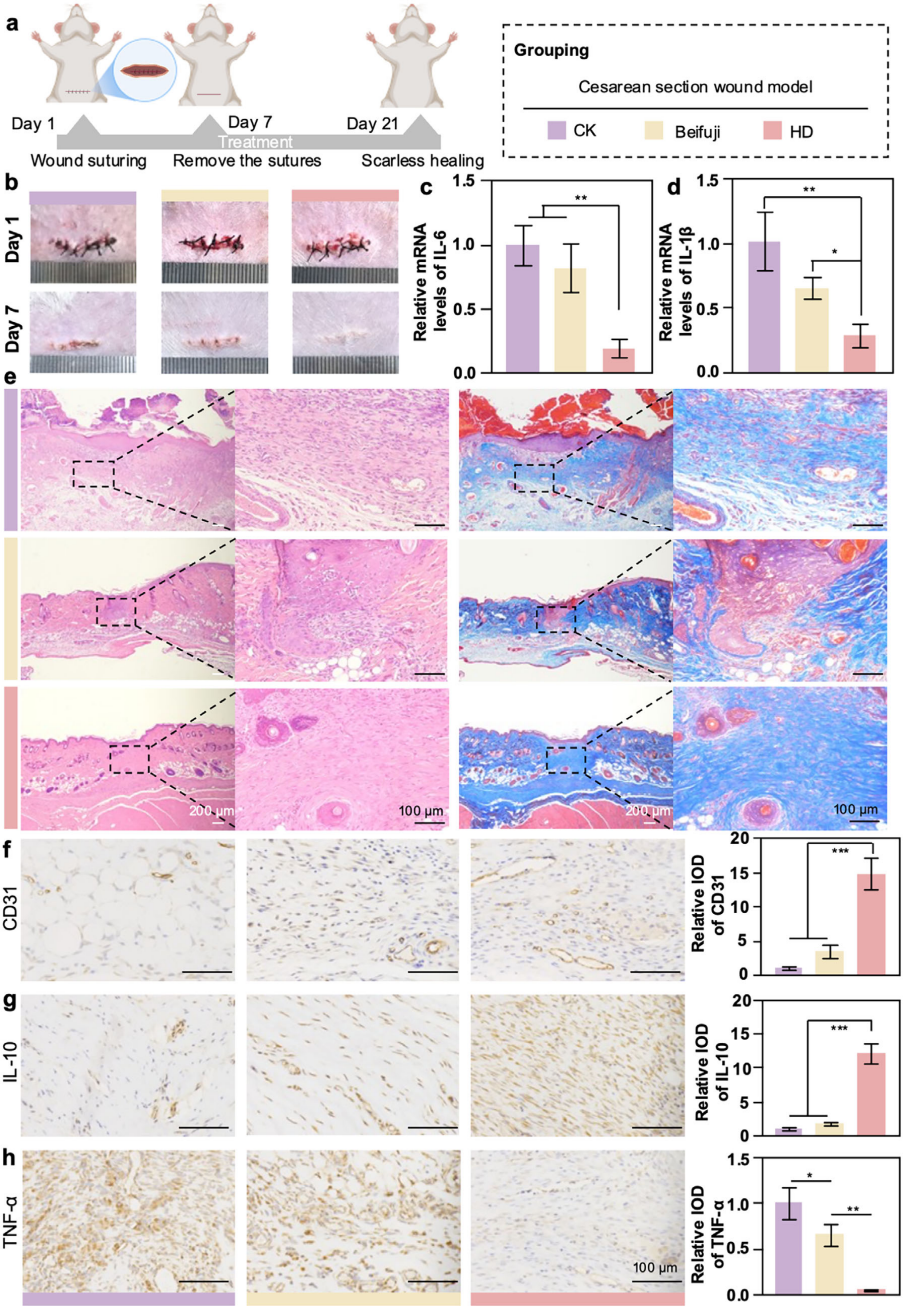
HD improves the early repair microenvironment in rat abdominal incisions. (a) Schematic illustration of the rat abdominal incision model and treatment protocol. (b) Representative photographs of wounds. Relative mRNA expression levels of (c) IL‐6 and (d) IL‐1β (*n* = 3). (e) Representative H&E and Masson's trichrome staining on day 7. Representative images and quantitative analyses of immunohistochemical staining for (f) CD31, (g) IL‐10, and (h) TNF‐α on day 7 (*n* = 3). Statistical significance was analyzed using one‐way ANOVA (^*^ *p* < 0.05, ^**^ *p* < 0.01, ^***^ *p* < 0.001). Error bars represent standard deviation.

The healing of abdominal incisions is often accompanied by excessive skin tension, which not only increases the risk of wound dehiscence but also promotes scar formation and abnormal tissue remodeling, thereby compromising the cosmetic outcome of healing [40]. To evaluate the therapeutic effect of HD on postoperative scar repair and to simulate wound healing following abdominal surgery, such as cesarean section, a rat abdominal wall incision model was established. The experimental design is illustrated in Figure 5a. After surgery, sutures were removed on postoperative day 7, and histological and molecular analyses were performed through postoperative day 21. At the time of suture removal, the incisions in all groups had largely closed. However, the CK group still exhibited obvious scar thickening and localized scar elevation, whereas the HD‐treated wounds showed a flatter surface with markedly reduced scar formation (Figure 5b). RT‐qPCR analysis further demonstrated that HD treatment significantly decreased the mRNA expression levels of IL‐6 and IL‐1β in wound tissues (Figure 5c,d), indicating effective attenuation of the postoperative inflammatory response.

Histological analysis further supported these findings. H&E staining revealed varying degrees of epidermal thickening, dermal disorganization, and inflammatory cell infiltration in both the CK and Beifuji groups. In contrast, the HD group exhibited a more intact skin architecture, with epidermal thickness approaching that of normal skin, markedly reduced inflammatory infiltration, and relatively complete regeneration of hair follicles and other skin appendages. Masson's trichrome staining showed abundant, densely packed, and disorganized collagen fibers in the CK group, whereas collagen fibers in the HD group were more regularly aligned, resulting in a tissue architecture that more closely resembled normal skin (Figure 5e). Immunohistochemical analysis further elucidated the biological effects of HD on the wound microenvironment. HD treatment significantly increased CD31 expression, indicating enhanced angiogenesis. Meanwhile, HD markedly upregulated the anti‐inflammatory cytokine IL‐10 while suppressing the expression of the pro‐inflammatory cytokine TNF‐α (Figure 5f–h), suggesting that HD facilitates inflammation resolution and establishes a regenerative microenvironment conducive to tissue repair. These findings further support that HD effectively attenuates postoperative scar formation in abdominal incisions by alleviating inflammation, enhancing angiogenesis, and promoting orderly collagen remodeling, ultimately improving the quality of skin repair.

### 2.5 Reduced Postoperative Scarring and Regeneration of Skin Appendages

**FIGURE 6:**
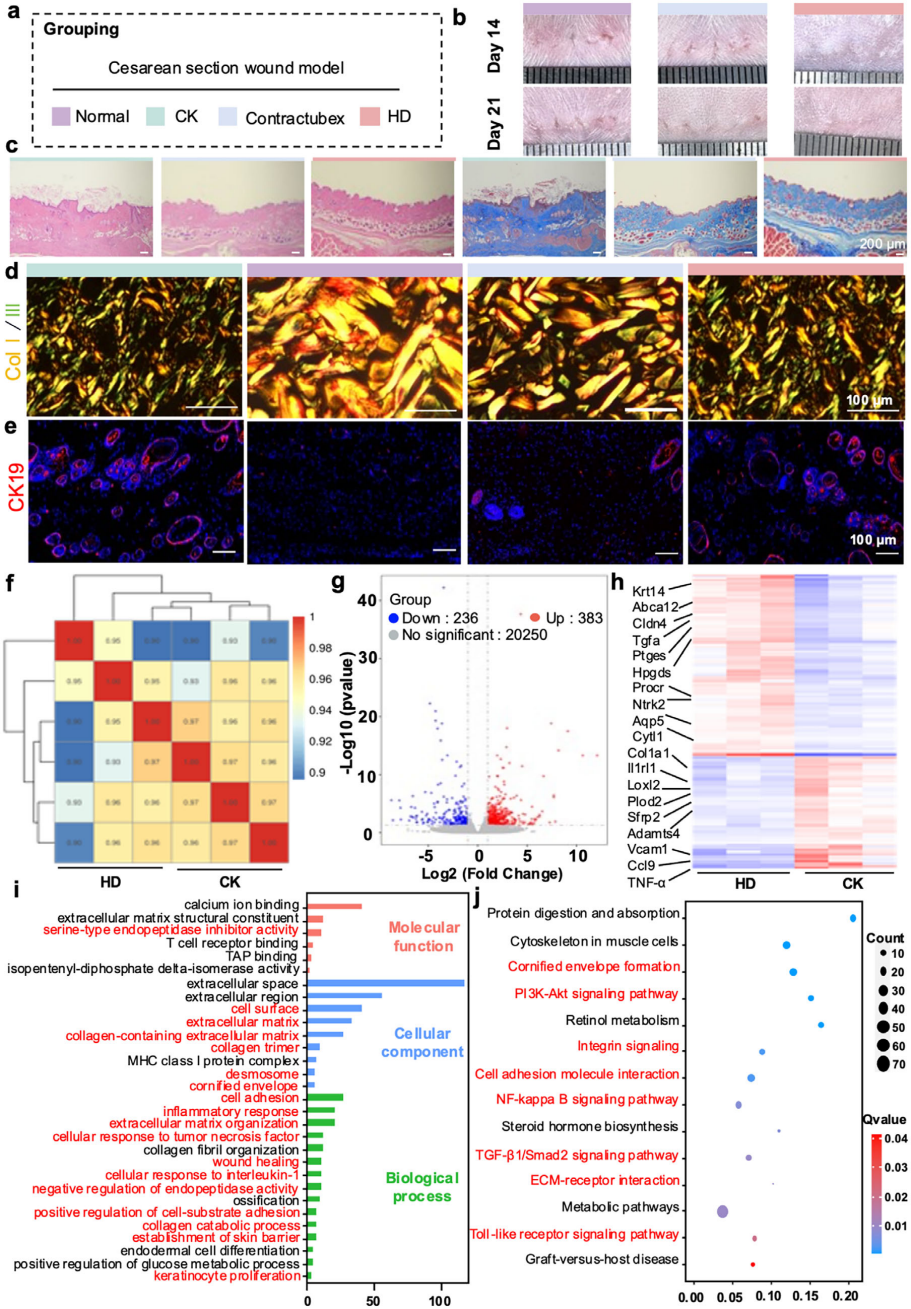
HD inhibits postoperative scar formation and regulates the expression of genes associated with wound repair. (a) Experimental design for postoperative scar treatment. (b) Representative photographs of postoperative wounds. (c) Representative H&E and Masson's trichrome staining on day 21. Representative (d) Sirius Red staining and (e) CK19 immunofluorescence staining on day 21. (f) Correlation analysis of transcriptomic datasets. (g) Volcano plot of differentially expressed genes. (h) Heatmap of differentially expressed genes. (i) GO enrichment analysis. (j) KEGG pathway enrichment analysis.

To further evaluate the regulatory effects of HD during postoperative scar maturation and tissue remodeling, Contractubex, a clinically widely used anti‐scarring agent, was selected as the positive control in this phase of the study. In our previous wound healing experiments, Befuju, a clinical wound‐healing product, served as the positive control for wound closure. However, because the present study focused on postoperative scar formation, Contractubex was deemed a more appropriate reference. Contractubex is a standardized anti‐scarring formulation containing onion extract, sodium heparin, and allantoin as its major active components. It exhibits anti‐inflammatory and anti‐fibrotic properties, regulates collagen remodeling, and has been widely used clinically for the treatment of hypertrophic and postoperative scars [41]. Therefore, it served as a clinically relevant benchmark for evaluating the anti‐scarring efficacy of HD. The experimental design is shown in Figure 6a. Rats were divided into Normal, CK, Contractubex, and HD groups, and scar tissues were systematically evaluated on postoperative days 14 and 21. Macroscopic observations showed that, following complete wound closure, the regenerated skin in the HD group more closely resembled normal skin. By day 14, wounds treated with HD exhibited better tissue elasticity, more uniform epidermal pigmentation, and less discoloration. By day 21, the regenerated epidermis displayed a color nearly identical to that of the surrounding normal skin, with almost no visible color difference. In contrast, obvious scar tissue and pigmentation abnormalities remained in both the CK and Contractubex groups (Figure 6b). qRT‐PCR results showed that HD treatment significantly upregulated the expression of the anti‐inflammatory cytokine IL‐10 while suppressing the expression of the pro‐inflammatory cytokine TNF‐α. Furthermore, analysis of fibrosis‐related genes revealed that HD significantly downregulated TGF‐β1 mRNA expression and upregulated TGF‐β3 mRNA expression, thereby increasing the TGF‐β3/TGF‐β1 ratio (Figure S9). These findings suggest that HD can inhibit excessive fibrosis and reduce the risk of scar formation.

Histological analysis further demonstrated the beneficial effects of HD on tissue regeneration. H&E staining revealed that the HD group developed a continuous and well‐organized epidermis with a flatter wound surface, exhibiting histological features closely resembling normal skin. More importantly, mature skin appendages, including hair follicles and associated structures, were regenerated in HD‐treated tissues, whereas appendage regeneration remained limited in the CK and Contractubex groups (Figure 6c and Figure S10). Since collagen remodeling is a critical determinant of scar quality, Sirius Red staining was performed to evaluate collagen composition within the regenerated tissue. The CK and Contractubex groups exhibited abundant COL‐I deposition with relatively low levels of COL‐III. In contrast, collagen in the HD group was predominantly composed of COL‐III, accompanied by markedly reduced COL‐I deposition, with collagen composition comparable to that of the Normal group (Figure 6d and Figure S11). Collagen fiber orientation analysis showed that collagen fibers in the CK group were randomly distributed, whereas both Contractubex and HD improved collagen alignment. Notably, the HD group exhibited a more organized collagen architecture, with a fiber orientation pattern closer to that of normal skin (Figure S12). These findings indicate that HD effectively promotes physiological collagen remodeling and reduces the risk of scar formation. To further evaluate tissue regeneration, immunofluorescence staining was performed using cytokeratin 19 (CK19) as a marker for hair follicle epithelial cells. CK19‐positive signals were significantly increased in the HD group, further confirming hair follicle regeneration (Figure 6e and Figure S13). Additionally, distinct β3‐tubulin‐positive nerve bundles were observed in regenerated tissues following HD treatment (Figures S14 and S15), suggesting that HD not only promotes the regeneration of skin appendages but also facilitates neural tissue reconstruction. The coordinated regeneration of skin appendages and nerve fibers suggests that HD‐mediated repair extends beyond simple wound closure and more closely resembles functional skin regeneration.

To systematically investigate the molecular mechanisms underlying HD‐mediated wound repair, whole‐transcriptome RNA sequencing was performed on regenerated wound tissues collected 21 days after treatment. The PBS‐treated group served as the control to identify the biological effects induced by HD. Correlation analysis showed that the R^2^ values among all biological replicates exceeded 0.90, indicating excellent reproducibility and reliability of the sequencing data (Figure 6f). Volcano plot analysis identified 383 significantly upregulated genes and 236 significantly downregulated genes in the HD group compared with the control group (Figure 6g). Functional enrichment analysis of differentially expressed genes revealed that HD significantly activates multiple biological processes associated with wound repair. Specifically, HD upregulates genes involved in epidermal regeneration, skin barrier formation, tissue repair, and immune regulation. Notably, Krt14, Abca12, and Cldn4 participate in basal keratinocyte proliferation, lipid transport, and tight junction formation, respectively, suggesting enhanced epidermal reconstruction and restoration of barrier function. Additionally, the increased expression of Tgfa, Procr, and Ntrk2 indicates enhanced cell proliferation, tissue regeneration, and vascular repair. Conversely, HD markedly suppresses the expression of genes associated with extracellular matrix remodeling and collagen deposition, including Col1a1, Loxl2, Plod2, Sfrp2, and Adamts4, as well as inflammation‐related genes such as Il1rl1, Vcam1, Ccl9, and Tnf, indicating that HD effectively attenuates persistent inflammation and pathological fibrosis (Figure 6h).

Gene Ontology (GO) analysis further demonstrated that the differentially expressed genes were primarily enriched in biological processes related to inflammatory regulation, extracellular matrix remodeling, collagen metabolism, and skin barrier reconstruction (Figure 6i). Kyoto Encyclopedia of Genes and Genomes (KEGG) pathway enrichment analysis revealed significant modulation of multiple signaling pathways involved in tissue regeneration, inflammation, and fibrosis following HD treatment (Figure 6j). Notably, the NF‐κB and Toll‐like receptor signaling pathways, which regulate inflammatory responses and innate immunity, were significantly enriched, consistent with the downregulation of inflammatory genes identified by transcriptomic analysis. Additionally, enrichment of the TGF‐β1/Smad2 signaling pathway and the ECM–receptor interaction pathway corresponded to the suppression of fibrosis‐related genes, including Col1a1, Loxl2, and Plod2. Meanwhile, activation of the PI3K–Akt, integrin signaling, and cell adhesion molecule interaction pathways supports enhanced cell migration, tissue remodeling, and re‐epithelialization. Furthermore, enrichment of the cornified envelope formation pathway suggests improved skin barrier maturation and functional recovery. These transcriptomic findings provide molecular evidence that HD coordinates inflammatory regulation, extracellular matrix remodeling, collagen homeostasis, and epidermal barrier reconstruction, thereby promoting high‐quality abdominal wound repair with reduced scar formation.

### 2.6 CD14 Engagement Suppresses NF‐κB, AKT, and Smad2 Signaling and Reprograms Macrophage Polarization

**FIGURE 7:**
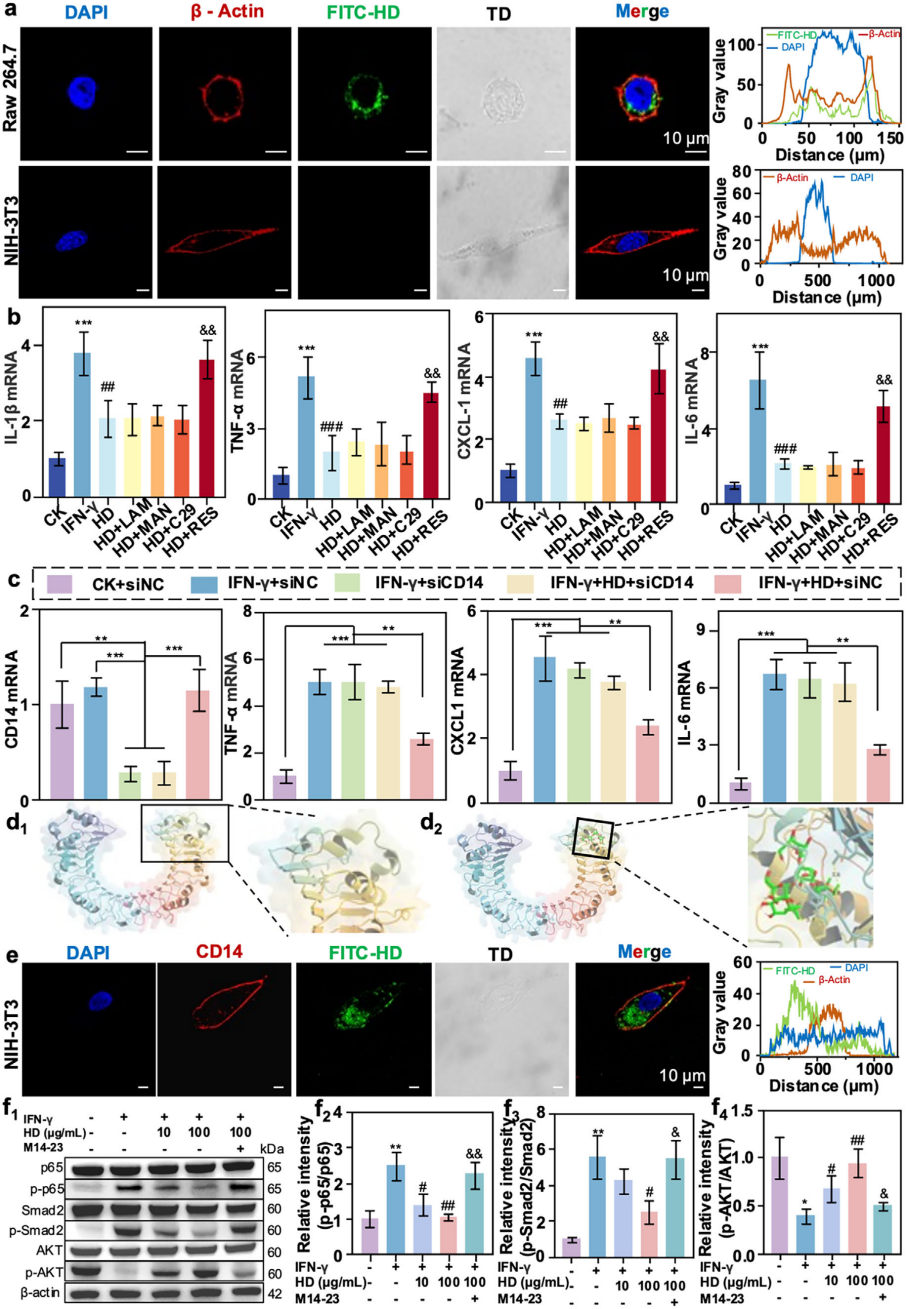
CD14‐dependent mechanism underlying the biological activity of HD. (a) Confocal fluorescence images showing the interaction of FITC‐labeled HD (green) with RAW 264.7 macrophages and NIH‐3T3 fibroblasts. Cell membranes were labeled with DiI (red), and nuclei were stained with DAPI (blue). (b) Effects of receptor blockade with laminarin (Dectin‐1 inhibitor), C29 (TLR2 inhibitor), mannan (DC‐SIGN inhibitor), and resatorvid (TLR4 inhibitor) on the anti‐inflammatory activity of HD in RAW 264.7 cells (*n* = 3). ^***^ *p* < 0.001 vs. the CK group. ^##^ *p* < 0.01, ^###^ *p* < 0.001 vs. the IFN‐γ group. && *p* < 0.01 vs. the HD group. (c) Effects of CD14 knockdown on HD‐mediated regulation of inflammatory cytokine expression under IFN‐γ stimulation (*n* = 3). Statistical significance was analyzed using one‐way ANOVA (^**^ *p* < 0.01, ^***^ *p* < 0.001). (d_1_) Three‐dimensional structure of CD14. (d_2_) Molecular docking analysis between HD and CD14. (e) Confocal fluorescence images showing FITC‐HD binding to NIH‐3T3 cells after CD14 overexpression. (f_1_) Representative western blot images showing p65 and p‐p65 in RAW 264.7 cells, Smad2 and p‐Smad2 in NIH‐3T3 cells, and AKT and p‐AKT in HUVECs. (f_2–4_) Quantification of Western blot results (*n* = 3). ^*^ *p* < 0.05, ^**^ *p* < 0.01 vs. the CK group. ^#^ *p* < 0.05, ^##^ *p* < 0.01 vs. the IFN‐γ group. & *p* < 0.05, && *p* < 0.01 vs. the HD_h_ group. Statistical significance was analyzed using one‐way ANOVA and an unpaired Student's t‐test. Error bars represent standard deviation.

To elucidate the cellular target of HD, FITC‐labeled HD was incubated with RAW 264.7 macrophages and NIH‐3T3 fibroblasts for 30 min (Figure 7a). Fluorescence microscopy revealed strong colocalization of FITC‐HD with the DiI‐labeled macrophage membrane, whereas almost no signal was detected in fibroblasts, suggesting preferential binding to macrophage surface receptors. Time‐course observations further showed rapid membrane association within 1–15 min followed by internalization (Figure S16), indicating specific receptor‐mediated recognition.

To identify the responsible receptor, membrane proteins were captured using HD‐coated microspheres and analyzed by liquid chromatography–tandem mass spectrometry (LC–MS/MS). Based on the mass spectrometry (MS) score and biological relevance, CD14 was identified as the most promising candidate receptor (Table S1). Blocking experiments were conducted using inhibitors targeting Dectin‐1 (laminarin), TLR2 (C29), DC‐SIGN (mannan), and TLR4 (resatorvid) (Figure 7b). HD significantly suppressed IFN‐γ–induced expression of IL‐1β, TNF‐α, CXCL1, and IL‐6; however, this anti‐inflammatory effect was partially reversed by resatorvid, implicating the CD14/TLR4 axis. As a classical pattern recognition receptor (PRR), CD14 can directly recognize polysaccharides. It cooperates with the TLR4–MD‐2 complex during ligand recognition and also regulates TLR4 internalization, making it a biologically plausible receptor for HD‐mediated signaling [42]. To confirm whether HD's therapeutic effects depend on CD14/TLR4 signaling, the antagonist IAXO‐101 was administered in a deep second‐degree burn model (Figure S17a). HD markedly accelerated wound closure, whereas CD14/TLR4 inhibition delayed healing; co‐administration with IAXO‐101 only partially restored the therapeutic effect (Figure S17b,c). qRT‐PCR further showed that HD significantly reduced IL‐6 and TNF‐α mRNA levels, an effect attenuated by IAXO‐101 (Figure S17d,e). These results indicate that intact CD14/TLR4 signaling is required for the full wound‐healing and anti‐inflammatory activity of HD.

To verify the functional importance of CD14, CD14 expression was knocked down in RAW 264.7 cells using small interfering RNA (siRNA). Under IFN‐γ stimulation, the expression of TNF‐α, CXCL1, and IL‐6 was markedly increased, whereas HD effectively suppressed their expression. This inhibitory effect was significantly attenuated following CD14 knockdown, indicating that the immunomodulatory activity of HD is largely CD14‐dependent (Figure 7c). Molecular docking analysis further confirmed a direct interaction between HD and CD14. Docking the three‐dimensional structure of HD with CD14 using AutoDock revealed a stable binding mode with a binding energy of −7.0 kcal/mol, indicating a favorable binding affinity (Figure 7d_1–2_). To further validate the essential role of CD14 in HD recognition, a mouse CD14 expression plasmid was transfected into NIH‐3T3 cells, which normally lack CD14 expression. Following transfection, NIH‐3T3 cells expressing mCherry‐labeled CD14 exhibited strong binding to FITC‐HD, whereas untransfected cells showed almost no FITC‐HD signal (Figure 7e). These findings further confirm the specific recognition of HD by CD14.

Given that CD14 is a key upstream regulator of innate immune signaling and transcriptomic analysis revealed significant enrichment of the NF‐κB, Toll‐like receptor, PI3K–AKT, and TGF‐β signaling pathways, the downstream signaling events mediated by HD were further investigated (Figure 7f_1_ and Figure S18). Western blot analysis showed that IFN‐γ stimulation markedly increased p65 phosphorylation in RAW264.7 macrophages, indicating activation of the NF‐κB pathway, whereas HD treatment significantly reduced p‐p65 levels (Figure 7f_2_), suggesting effective suppression of NF‐κB‐mediated inflammatory signaling [43]. This inhibitory effect was largely abolished by CD14 neutralization, indicating that HD regulates NF‐κB activation in a CD14‐dependent manner. Consistently, enzyme‐linked immunosorbent assay (ELISA) analysis demonstrated that HD dose‐dependently reduced the secretion of the pro‐inflammatory cytokines TNF‐α and IL‐6 while significantly increasing IL‐10 production (Figure S19). These immunomodulatory effects were markedly attenuated following CD14 blockade, further supporting the essential role of CD14 in HD‐mediated macrophage reprogramming. In fibroblasts, exposure to the HD‐regulated inflammatory microenvironment markedly reduced Smad2 phosphorylation (Figure 7f_3_). Correspondingly, the expression of the fibrosis‐associated genes collagen type I alpha 1 chain (COL1A1) and α‐SMA was significantly decreased, whereas collagen type III alpha 1 chain (COL3A1) expression was increased (Figure S19). Since Smad2 is a key downstream mediator of TGF‐β signaling that drives fibrotic remodeling and excessive collagen deposition [44], these findings suggest that HD attenuates fibroblast activation by reshaping the macrophage inflammatory microenvironment. These regulatory effects were partially reversed following CD14 blockade. Likewise, exposure to the HD‐regulated inflammatory microenvironment significantly restored AKT phosphorylation in endothelial cells (Figure 7f_4_), whereas this pro‐angiogenic response was diminished after CD14 neutralization. Restoration of AKT signaling is closely associated with enhanced endothelial cell survival, migration, and angiogenesis [45]. These findings demonstrate that HD suppresses NF‐κB‐mediated inflammatory activation through CD14, thereby remodeling the inflammatory microenvironment to inhibit TGF‐β/Smad2‐dependent fibroblast activation while promoting AKT‐mediated angiogenic responses, ultimately coordinating inflammation resolution, angiogenesis, and extracellular matrix remodeling during wound repair.

### 2.7 HD Promotes the Formation of a Regenerative Wound Microenvironment

**FIGURE 8:**
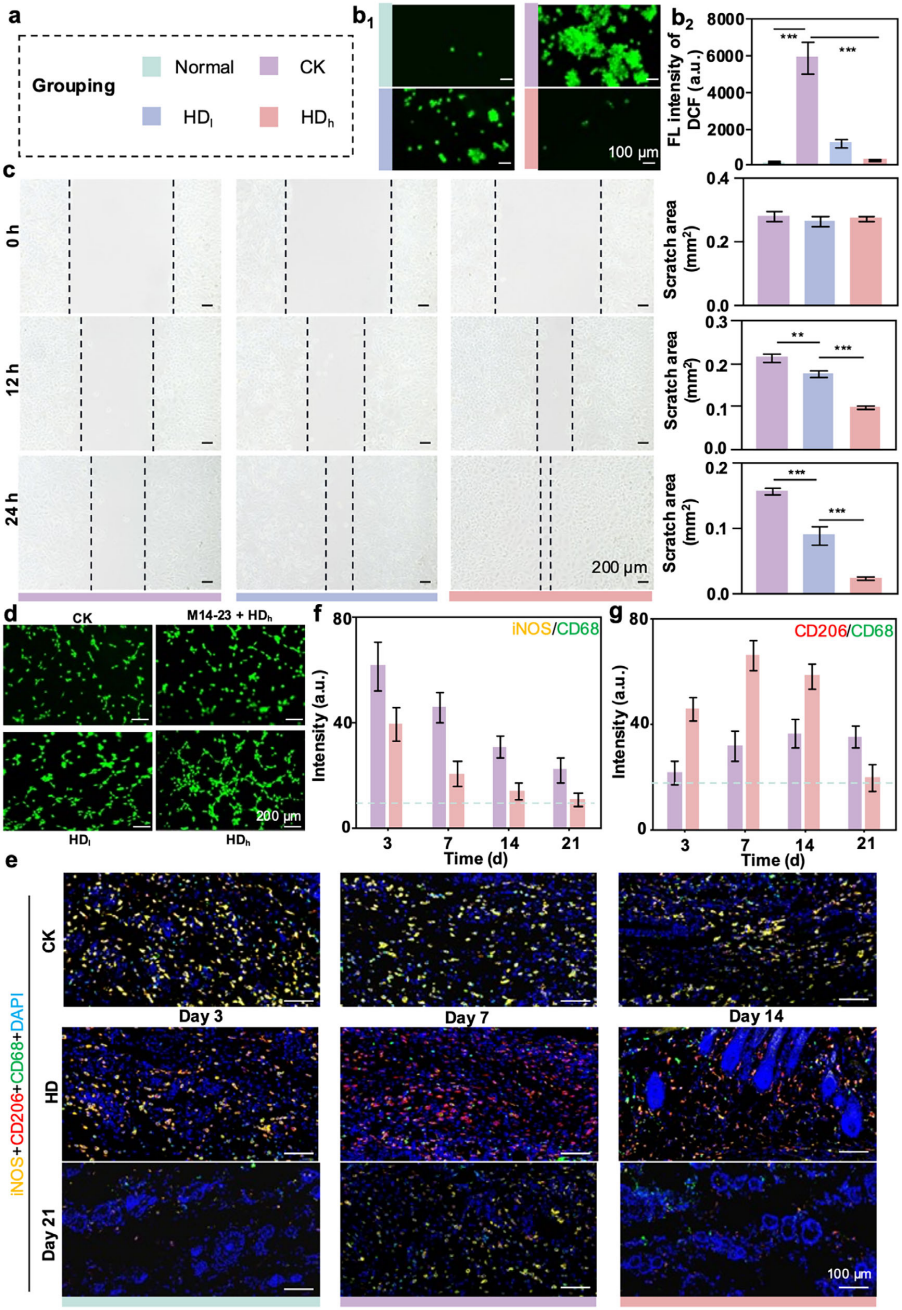
HD promotes the establishment of a regenerative wound microenvironment. (a) Schematic illustration of the experimental design. (b_1_) Representative fluorescence images showing intracellular ROS levels in RAW 264.7 cells following HD treatment. (b_2_) Quantification of intracellular ROS fluorescence intensity (*n* = 3). (c) Representative scratch wound images and quantitative analysis of NIH‐3T3 cell migration (*n* = 3). (d) Representative tube formation images and quantitative analysis of HUVEC angiogenesis following HD treatment (*n* = 3). (e) Representative immunofluorescence images of iNOS, CD206 and CD68 staining in rat abdominal wound tissues. Quantification of (f) iNOS/CD68 cells and (g) CD206/CD68 cells. Statistical significance was analyzed using one‐way ANOVA (^**^ *p* < 0.01, ^***^ *p* < 0.001); ns indicates no significant difference. Error bars represent standard deviation.

The proliferative phase of wound healing is a critical stage for tissue reconstruction, and its successful progression depends on the resolution of oxidative stress, cell migration, angiogenesis, and the coordinated regulation of the inflammatory microenvironment. Based on findings from animal studies, we further systematically evaluated the regulatory effects of HD on the regenerative wound microenvironment (Figure 8a). Excessive accumulation of ROS in the wound microenvironment can damage the extracellular matrix, impair angiogenesis, disrupt tissue integrity, and ultimately delay wound healing [46]. Intracellular ROS levels were assessed in RAW 264.7 cells using the DCFH‐DA fluorescent probe. As shown in Figure 8b_1–2_, H_2_O_2_ stimulation markedly increased intracellular ROS accumulation, whereas HD treatment significantly reduced ROS fluorescence intensity in a dose‐dependent manner, indicating that HD effectively alleviates oxidative stress and creates a more favorable microenvironment for tissue regeneration. We next evaluated the effects of HD on key cellular events involved in wound repair. Scratch assay results showed that HD significantly promoted the migration of NIH‐3T3 cells compared with the control group, with the high‐dose HD group exhibiting the greatest enhancement (Figure 8c). In addition, Matrigel tube formation assays demonstrated that the HD‐regulated inflammatory microenvironment significantly enhanced the angiogenic capacity of HUVECs, resulting in a more extensive and well‐organized capillary‐like network than that observed in the IFN‐γ group (Figure 8d). This pro‐angiogenic effect was markedly attenuated following CD14 neutralization, consistent with the changes in AKT phosphorylation (Figure 8d and Figure S20). These findings indicate that HD effectively promotes angiogenesis, thereby facilitating the delivery of oxygen and nutrients required for tissue regeneration.

In addition to promoting tissue regeneration, HD also exhibited pronounced immunomodulatory activity. The transition of macrophages from the pro‐inflammatory M1 phenotype to the reparative M2 phenotype is widely recognized as a hallmark of the shift from the inflammatory phase to the proliferative phase of wound healing [47]. To further investigate the dynamic changes in macrophage polarization during rat abdominal wound healing, immunofluorescence co‐staining was performed on wound tissues collected at postoperative days 3, 7, 14, and 21 (Figure 8e). HD treatment markedly reduced the proportion of M1 macrophages (iNOS/CD68) while increasing the proportion of M2 macrophages (CD206/CD68) throughout the healing process. Quantitative analysis further confirmed a progressive decrease in M1 macrophages accompanied by a corresponding increase in M2 macrophages following HD treatment (Figure 8f,g). These findings were corroborated in the mouse wound model, where HD accelerated the iNOS to Arg‐1 phenotypic switch, with Arg‐1 peaking at day 7 (Figure S21). qRT‐PCR further confirmed that HD suppressed IL‐6 and TNF‐α mRNA levels, with maximal reduction at day 3. Together, these findings indicate that HD effectively suppresses excessive inflammation while promoting the timely resolution of the inflammatory response. Combined with the receptor‐blocking experiments described above, these results further indicate that this phenotypic transition is mediated predominantly through CD14‐dependent signaling, as blockade of CD14 attenuated HD‐induced M2 polarization and partially restored the pro‐inflammatory M1 phenotype, thereby compromising the establishment of a pro‐regenerative immune microenvironment favorable for wound repair.

## 3 Discussion

**FIGURE 9:**
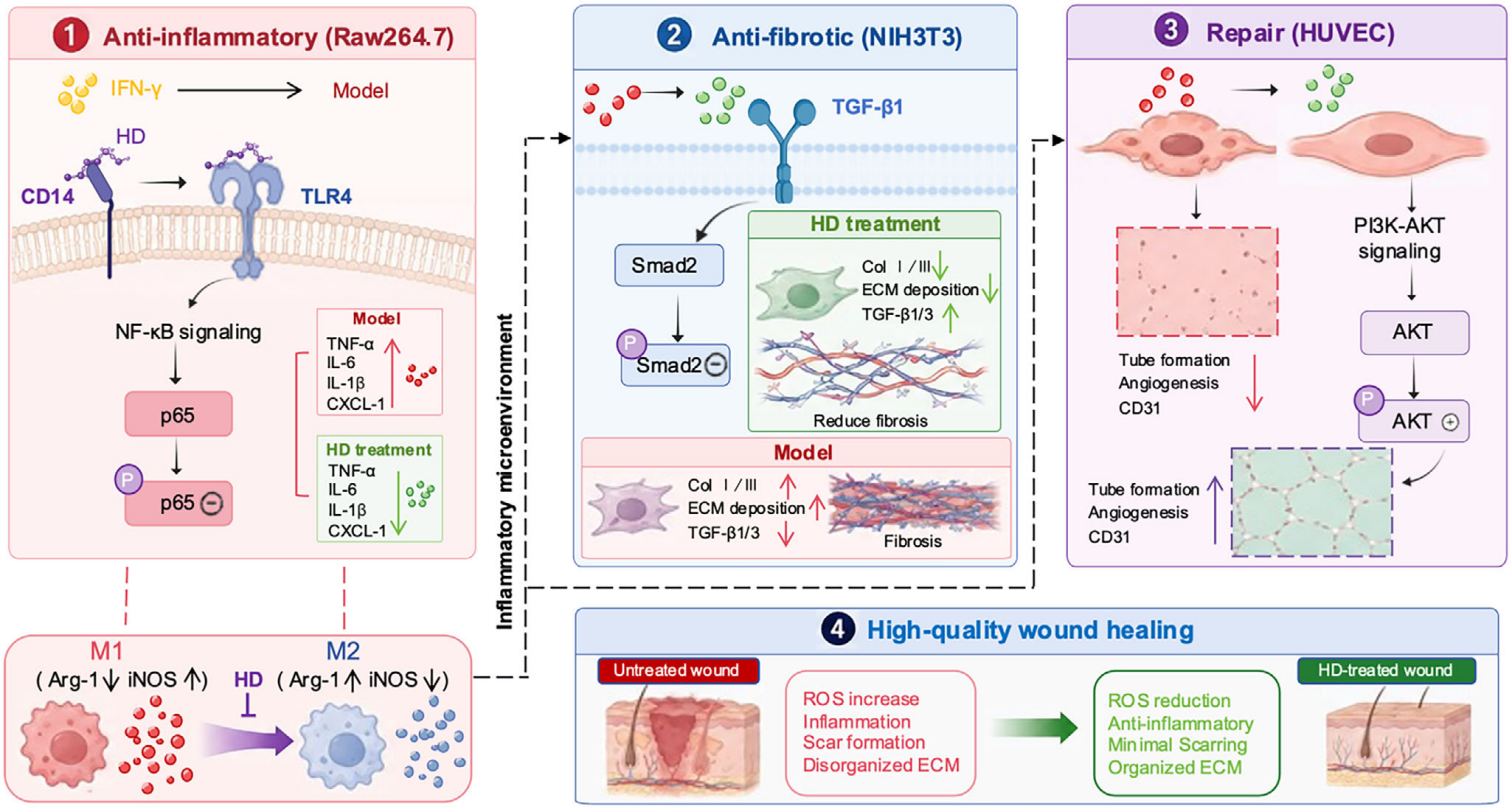
Mechanism underlying HD‐mediated promotion of high‐quality wound healing.

This study reveals that CD14, a pattern recognition receptor traditionally known for pathogen sensing, serves as a therapeutically actionable target for macrophage reprogramming in wound repair. Using a combination of affinity capture, receptor blockade, and genetic manipulation, we identified CD14 as the primary recognition receptor for the microbial polysaccharide HD. When formulated as an in situ self‐gelling powder, HD promotes scar‐reducing regenerative wound healing in three preclinical models. Mechanistically, HD engagement of CD14 suppresses NF‐κB‐driven inflammation in macrophages, and the secretome of HD‐reprogrammed macrophages subsequently enhances AKT‐dependent angiogenesis in endothelial cells while indirectly attenuating TGF‐β/Smad2‐driven fibrosis in fibroblasts, thereby remodeling the wound microenvironment. As summarized in Figure 9, this CD14‐associated signaling network establishes a paradigm in which a single natural polysaccharide coordinates multicellular repair processes through a single molecular target, offering both mechanistic insight and translational simplicity.

Pathological scarring is not merely a passive outcome of tissue repair but an active consequence of sustained macrophage‐mediated inflammation that drives fibroblast hyperactivation and excessive ECM deposition [48]. Current anti‐scarring strategies primarily target fibroblasts or established scars rather than intercepting the pathogenic process at its origin. Our findings establish a fundamentally different approach. CD14 has long been recognized for its role in pathogen sensing as a co‐receptor for TLR4 [49], but its potential as a target for regenerative immunomodulation has remained largely unexplored. Notably, previous studies have demonstrated that CD14 mediates macrophage migration during wound healing, and macrophages from CD14‐deficient mice exhibit impaired migratory capacity, highlighting an essential role for CD14 in tissue repair [50]. These findings are consistent with our identification of CD14 as a functional receptor for HD and support the biological relevance of the HD–CD14 axis in regulating wound healing. By demonstrating that a naturally derived polysaccharide engages CD14 to modulate host immune responses, our study expands the current understanding of CD14 beyond its established role in pathogen recognition and innate immune defense, revealing its potential as a therapeutic target for immune reprogramming during tissue regeneration.

The multicellular coordination achieved by HD is particularly noteworthy. Rather than directly suppressing fibroblast activity, which could impair normal tissue remodeling, HD acts upstream by reprogramming macrophages that regulate fibroblast behavior. This approach aligns with the emerging concept that macrophages serve as central “conductors” of the repair process, and that therapeutic targeting of macrophage polarization can redirect the entire healing trajectory [51]. The indirect inhibition of TGF‐β/Smad2 signaling through the HD‐remodeled macrophage secretome is especially significant, as TGF‐β is a master regulator of fibrotic remodeling via both canonical (Smad‐dependent) and non‐canonical pathways [52]. Aberrant TGF‐β/Smad signaling has been implicated in hypertrophic scars and keloids, where it promotes excessive collagen production and fibroblast hyperproliferation [44]. By intercepting this fibrotic cascade at the macrophage level, rather than attempting to suppress fibroblast activity after fibrosis has already been initiated, HD prevents the establishment of a pro‐fibrotic microenvironment from the earliest stages of healing. This temporal advantage distinguishes HD from conventional anti‐scarring agents, which are typically applied after epithelialization and act on already‐formed scars.

The NF‐κB pathway, which HD suppresses through CD14 engagement, is a key driver of pro‐inflammatory macrophage polarization; its sustained activation maintains the M1 phenotype and chronic inflammation [53]. Following HD‐mediated macrophage reprogramming, activation of PI3K/AKT signaling supports endothelial proliferation, angiogenesis, and vascular reconstruction, whereas attenuation of TGF‐β/Smad2 signaling limits excessive fibroblast activation and collagen deposition. The coordinated regulation of these pathways highlights the central role of macrophages in integrating inflammatory resolution, tissue regeneration, and fibrotic remodeling. This coordinated modulation of inflammation, angiogenesis, and fibrosis enables HD to reshape the wound microenvironment and promote regenerative healing with reduced scar formation [54]. By regulating multiple stages of repair through a macrophage‐centered mechanism, HD reshapes the wound microenvironment and promotes regenerative healing with reduced scar formation. Notably, this mechanism differs from those of most naturally derived polysaccharides currently used for wound repair. Cellulose and hemicellulose primarily facilitate wound healing by providing extracellular matrix‐like structural support, maintaining a favorable microenvironment for cell adhesion and tissue regeneration [55, 56]. Starch‐based biomaterials mainly promote wound repair through rapid hemostasis and temporary wound coverage [57]. Alginate undergoes ion‐mediated gelation upon contact with wound exudates, forming a moist hydrogel that absorbs excess exudates, supports granulation tissue formation, and facilitates re‐epithelialization [58]. Dextran has been shown to promote angiogenesis, collagen deposition, and tissue remodeling and is frequently employed as a hydrogel matrix or drug‐delivery platform [57]. Hyaluronic acid, a major component of the extracellular matrix, regulates cell migration, tissue hydration, extracellular matrix remodeling, and angiogenesis through interactions with cell‐surface receptors such as CD44 [59]. Nevertheless, the wound‐healing activities of these polysaccharides are largely attributed to their physicochemical characteristics or indirect biological effects, and their therapeutic performance often depends on chemical modification, functionalization, or incorporation of exogenous bioactive molecules. In contrast, HD intrinsically targets the innate immune receptor CD14, thereby initiating a receptor‐defined signaling cascade that coordinately regulates inflammation, angiogenesis, and fibrosis. Unlike conventional wound‐healing strategies that rely on the combinatorial delivery of multiple bioactive agents, including anti‐inflammatory, pro‐angiogenic, and anti‐fibrotic factors, HD, as a single naturally derived polysaccharide, simultaneously orchestrates multiple stages of tissue repair through a unified CD14‐dependent mechanism [60, 61]. This receptor‐guided, multi‐functional mode of action not only distinguishes HD from existing polysaccharide‐based wound dressings but also simplifies material design while maintaining broad therapeutic efficacy. Furthermore, its in situ self‐gelling powder formulation enables rapid gelation upon contact with wound exudates or blood and immediate adaptation to irregular wound surfaces without pre‐hydration or complex handling, thereby enhancing its translational potential for emergency care and point‐of‐care applications [62].

Collectively, this study establishes a CD14‐targeting, in situ self‐gelling polysaccharide that actively reprograms the wound microenvironment through a macrophage‐centered mechanism, achieving coordinated regulation of inflammation, angiogenesis, and fibrosis. Beginning with CD14 engagement on macrophages, HD suppresses NF‐κB signaling and subsequently orchestrates downstream regenerative responses, ultimately directing wound healing toward tissue regeneration with reduced scar formation rather than passive repair. Beyond the development of an advanced wound dressing, this work reveals that naturally derived polysaccharides can function as precise immunomodulatory molecules through endogenous receptor engagement, providing a conceptual paradigm for designing next‐generation regenerative biomaterials via receptor‐guided immune modulation. These findings not only advance the mechanistic understanding of polysaccharide‐mediated tissue regeneration but also establish a conceptual framework for designing next‐generation biomaterials through precise receptor‐guided immunomodulation of the wound microenvironment.

## 4 Conclusions

In summary, this study developed a microbial polysaccharide‐derived, in situ self‐gelling powder dressing based on HD that rapidly forms an adhesive gel in the wound environment and effectively promotes high‐quality skin repair. In models of deep second‐degree burns, hypertrophic scars, and high‐tension incisions, HD accelerated wound closure, reduced excessive inflammation and fibrosis, improved collagen remodeling, and promoted the regeneration of skin appendages, blood vessels, and neural structures. Mechanistically, HD functions as an immunoregulatory biomaterial by targeting CD14 on macrophages and reshaping the macrophage‐mediated wound microenvironment. Through macrophage phenotype reprogramming and paracrine regulation, HD suppresses inflammatory signaling, enhances endothelial AKT phosphorylation to promote angiogenesis, and reduces fibroblast Smad2 phosphorylation to attenuate fibrotic remodeling. These findings reveal a macrophage‐centered mechanism by which natural microbial polysaccharides coordinate inflammation resolution, tissue regeneration, and scar inhibition, providing a promising strategy for developing next‐generation wound dressings capable of achieving rapid healing with minimal scar formation.

## 5 Experimental Section

### 5.1 Animal Ethics Statement

Male C57BL/6 mice (7–8 weeks old, 20–24 g), male New Zealand rabbits (12–14 weeks old, 2.8–3.5 kg), and female Sprague–Dawley rats (8–10 weeks old, 220–260 g) were used in this study. All animal experiments were conducted in accordance with the ARRIVE guidelines and were approved by the Institutional Animal Care and Use Committee of Nanjing University of Science and Technology (IACUC‐NJUST‐2024‐1105).

### 5.2 Statistical Analysis

All in vitro experiments were performed in at least three independent biological experiments unless otherwise stated. Where applicable, technical replicates were averaged to obtain a single value for each biological replicate and were not considered independent replicates for statistical analysis. Data are presented as mean ± standard deviation (SD). Statistical analyses were conducted using GraphPad Prism 9 and ImageJ software. Student's t‐test was used for comparisons between two groups, while one‐way ANOVA followed by Tukey's post hoc test was applied for multiple‐group comparisons. Statistical significance was defined as *p* < 0.05 (^*^), *p* < 0.01 (^**^), and *p* < 0.001 (^***^), while ns indicates no significant difference.

## Author Contributions

R.F. conceived the idea, designed the research, analyzed the data, and wrote the manuscript. S.M.C. and Q.Z.L participated in the experiment and figure revision. W.W.N and X.X contributed to data collection and analysis. J.F.Z. supervised and managed the research work. All authors discussed the results and contributed to the writing and revision of the manuscript.

## Conflicts of Interest

The authors declare no conflicts of interest.

## Supporting information




**Supporting File 1**: advs77602‐sup‐0001‐SuppMat.docx.

**Supporting File 2**: advs77602‐sup‐0001‐S1.xlsx.

## Data Availability

The data that support the findings of this study are available from the corresponding author upon reasonable request.
