# Supplementary material for: A CD14‐Targeting In Situ Hydrogel Directs Macrophage Reprogramming to Minimize Scar Formation
Source: Adv Sci (Weinh). 2026 Sep 6:e77602. Online ahead of print. doi: 10.1002/advs.77602 (PMC13547538; doi:10.1002/advs.77602)
Supplement: Supplementary file 1 [file ADVS-9999-e77602-s002.docx]

Supporting Information

**A CD14-Targeting in Situ Hydrogel Directs Macrophage Reprogramming to Minimize Scar Formation**

*Rui Fang ^a,b^, Simeng Chen ^a,b^, Qiaozhen Liu ^a,b^, Wenwen Ni ^a,b^, Xi Xu ^a,b^, Jianfa Zhang ^a,b^**

^a^ Center for Molecular Metabolism, Nanjing University of Science & Technology

^b^ Key Laboratory of Metabolic Engineering and Biosynthesis Technology, Ministry of Industry and Information Technology

***** Address correspondence to:

Jianfa Zhang

Center for Molecular Metabolism, Nanjing University of Science & Technology, 200 Xiaolingwei Street, Nanjing, 210094, China

E-mail: [jfzhang@mail.njust.edu.cn](mailto:jfzhang@mail.njust.edu.cn)

Keywords: Hemoadhican; CD14; Macrophage reprogramming; Immune microenvironment; Minimal-scarring wound repair

1. **Supplementary Experiment**

*1.1 Materials*

ROS assay kits were obtained from the Jiancheng Bioengineering Institute (Nanjing, China). The Cell Counting Kit-8 (CCK-8) assay kit was purchased from MedChemExpress LLC (Shanghai, China). BCA protein quantification kits, ROS assay kits, and DiI red fluorescent dye were acquired from Beyotime Institute of Biotechnology (Shanghai, China). Laminarin and mannan were purchased from Sigma-Aldrich (St. Louis, MO, USA), while C29 and resatorvid were obtained from MedChemExpress (Shanghai, China). IAXO-101 was purchased from Innaxon (UK). Lipofectamine RNAiMAX reagent was purchased from Beijing Duona Biomedical Technology Co., Ltd. (Beijing, China). Antibodies against β-actin were obtained from Abcam (Cambridge, UK), and antibodies against p65 and phosphorylated p65 (p-p65) were purchased from Santa Cruz Biotechnology (Dallas, TX, USA). Antibodies against AKT, phosphorylated AKT (p-AKT), Smad2, and phosphorylated Smad2 (p-Smad2) were purchased from Cell Signaling Technology (CST, Danvers, MA, USA). An anti-mouse CD14 blocking antibody (clone M14-23, rat IgG2a) was purchased from BioLegend (San Diego, CA, USA). The mouse embryonic fibroblast cell line (NIH-3T3), human umbilical vein endothelial cells (HUVECs), and murine macrophage cell line RAW 264.7 were obtained from the China Center for Type Culture Collection (CCTCC, Wuhan, China). Dulbecco’s Modified Eagle Medium (DMEM), fetal bovine serum (FBS), and penicillin–streptomycin (P/S) were purchased from Thermo Fisher Scientific Inc. (Waltham, MA, USA). Dermlin inorganic wound dressing, Beifuji gel, and Contractubex gel were obtained from a commercial supplier. Anticoagulated whole blood was prepared by mixing freshly collected rabbit blood with 3.8% sodium citrate solution at a ratio of 9:1 (v/v). All reagents were of analytical grade and used without further purification. All water used in the experiments was deionized.

*1.2 Preparation of HD Powder*

The HD polysaccharide was extracted and purified from the fermentation broth following the procedures outlined in our previous publication [1]. Briefly, the bacterial strain was inoculated into 50 mL of culture medium (pH 7.0) and incubated at 28 °C with shaking at 220 rpm for 72 h. After fermentation, the culture broth underwent purification processes to obtain HD polysaccharide with a reported purity of 99.9%. The purified product was then dried in an oven at 65 °C until a constant weight was achieved, followed by grinding into a fine powder for subsequent experiments.

*1.3 Morphological Characterization and Gelation Behavior of HD Powder*

To evaluate the liquid absorption and in situ gelation behavior of HD powder, 300 μL of stained phosphate-buffered saline (PBS) or anticoagulated whole blood was added to an equal mass of HD powder. The liquid absorption and gel formation processes were observed for 3 min, and images were captured at predetermined time points to document the morphological changes. The self-healing behavior of the resulting gels was also evaluated.

The gelation time of HD powder was determined using the vial inversion method. Briefly, HD powder was mixed with either PBS or fresh whole blood in glass vials and incubated at 37 °C. The vials were gently inverted every 5 second, and the gelation time was defined as the time required for the mixture to completely lose fluidity upon vial inversion. Each measurement was performed in triplicate.

The morphology and microstructure of HD powder, PBS-induced HD gels, and blood-induced HD gels were characterized using scanning electron microscopy (SEM; GeminiSEM 300, ZEISS). All samples were freeze-dried, sputter-coated with gold for 45 s at a current of 10 mA using a Quorum SC7620 sputter coater, and imaged at an accelerating voltage of 3 kV. The particle size of HD powder was analyzed from SEM images using ImageJ software by measuring at least 100 particles. The porosity of the freeze-dried HD gels was determined by the ethanol displacement method after immersion in absolute ethanol for 30 min and calculated according to a previously reported method [2].

*1.4 In vitro degradation experiments*

The in vitro degradation behavior of HD powder was evaluated by monitoring the change in dry weight after immersion in PBS (pH 7.4). Briefly, HD powder with a known initial dry weight (m_0_) was immersed in 10 mL of PBS and incubated at 37 °C. At predetermined time points, the samples were collected, freeze-dried to a constant weight, and weighed to determine the remaining dry mass (m_t_). The PBS solution was refreshed daily throughout the degradation period. The degradation ratio of HD powder was calculated using Equation (1):

$Weight loss=\frac{m_{0}-m_{t}}{m_{0}} \times100 \%$ (1)

where m_0_ is the initial dry weight of the HD sample, and m_t_ is the remaining dry weight at a specified time point.

*1.5 Young’s Modulus of HD Gels*

The Young's modulus of HD gels was determined from the compressive stress–strain curves. HD powder was mixed with either PBS or fresh whole blood and incubated at 37 °C until complete gelation. The resulting gels were prepared as cylindrical specimens with identical dimensions. Mechanical testing was performed using an electronic universal testing machine (ETM503A, WANCE, China). The samples were compressed at a crosshead speed of 2 mm min⁻¹, and the compressive stress–strain curves were recorded. The Young's modulus was calculated from the initial linear elastic region of the stress–strain curves.

*1.6 Rheological Testing*

The rheological properties of the gel samples were evaluated using a rotational rheometer (Physica MCR 101, Anton Paar) to characterize their viscoelastic behavior. A parallel-plate geometry (PP-50, 1 mm gap) was employed for all measurements, which were conducted at 37 °C. Alternating strain sweep tests were performed by switching between low strain (0.1%) and high strain (100%) to assess the structural stability and self-recovery capability of the gels. Frequency sweep measurements (0.1–100 Hz) were then carried out at a constant strain of 0.1% to determine the storage modulus (G′) and loss modulus (G″). Additionally, shear rate sweep tests (0.1–100 s⁻¹) were conducted at 37 °C under a constant strain of 0.1% to evaluate the shear-thinning behavior and viscosity changes of the gels.

*1.7 Tissue Adhesion Test*

Pig skin was used to evaluate the tissue adhesion properties of HD powder. The skin was cut into rectangular tissue patches (10 mm × 30 mm). HD powder was uniformly applied to the tissue surface, followed by the addition of PBS or anticoagulated whole blood at room temperature to induce in situ gel formation. The effective contact area between the two tissue patches was fixed at 10 mm × 10 mm.

1.7.1 Interfacial Toughness Test

The interfacial fracture toughness of HD on wet tissue surfaces was evaluated using a 180° peel test at a crosshead speed of 50 mm/min, following the ASTM F2256 standard. The interfacial toughness was calculated using Eq. (1):

$Interfacial toughness = \frac{2F}{W}$ (Eq. 1)

where F represents the steady-state plateau force, and W denotes the overlap width of the HD sample (n = 3).

1.7.2 Lap Shear Test

After equilibration in a humid environment at 37 °C for 1 h, lap shear tests were performed according to ASTM F2255 at a loading rate of 50 mm/min. Shear strength was calculated by dividing the maximum load by the adhesion area using Eq. (2):

$Shear strength = \frac{F}{A}$ (Eq. 2)

Where F is the maximum load, and A is the effective contact area between the HD gel and the tissue (n = 3).

*1.8 Biocompatibility Assessment*

The cytocompatibility of HD was evaluated by measuring the viability of RAW 264.7 cells using a CCK-8 assay, following the manufacturer's instructions. The hemocompatibility of HD powder was assessed through a hemolysis assay, based on the method described by Liu et al.[3].

*1.9 In vivo biodistribution and cellular infiltration of HD*

FITC-labeled HD (FITC-HD) was prepared according to the method described by Dong et al. and subcutaneously implanted into the backs of C57BL/6 mice (7–8 weeks old, weighing 20–24 g) under isoflurane anesthesia [9]. At 12, 24, 48, 72, and 96 h post-implantation, the mice were euthanized, and whole blood was collected. Blood samples were centrifuged at 3,500 rpm for 20 minutes to obtain plasma (n = 3 per group). Subsequently, the subcutaneous tissue at the implantation site and its surrounding area, as well as the heart, liver, spleen, lungs, and kidneys, were harvested. Each tissue was thoroughly rinsed with PBS, surface fluid was blotted dry, and the tissues were weighed. Nine times the tissue volume (w/v) of PBS was added for homogenization, followed by centrifugation at 3,500 rpm for 10 minutes. A 200-μL aliquot of plasma or the supernatant from the tissue homogenate was taken and diluted with PBS to a final volume of 2.5 mL. Fluorescence intensity of the plasma and tissue homogenates was measured using a fluorescence spectrophotometer (excitation wavelength 495 nm, emission wavelength 525 nm, slit width 2 nm). Plasma and tissue samples from untreated mice were analyzed under the same conditions; their fluorescence intensities were used as background values and subtracted prior to data analysis. Tissue samples from the implantation site, including the implant material and surrounding subcutaneous tissue, were collected at various time points and prepared as frozen sections. Cell nuclei were stained with DAPI, while the implanted HD was visualized using its intrinsic FITC fluorescence. Fluorescence images were acquired using an inverted fluorescence microscope (Ti2, Nikon) to assess cellular infiltration of the implanted HD.

*1.10 Application of HD Powder in Burn Wound Repair*

A deep second-degree burn model was established in mice as previously described [4-5]. Briefly, male C57BL/6 mice (7–8 weeks old, weighing 20–24 g) were anesthetized and shaved. An 8-mm-diameter copper rod was heated in boiling water and applied to the dorsal skin for 10 s to induce deep second-degree burn wounds. Twenty-four hours after injury, the necrotic tissue was surgically debrided using sterile scissors, and the animals were randomly assigned to the HD powder group, PBS group, or Dermlin group (n = 9 per group). The corresponding treatments were administered for 14 days, during which wound morphology and wound pH were monitored at predetermined time points. Wound tissues were collected on days 3, 7, and 14 for immunofluorescence co-localization and RT-qPCR analyses of macrophage M1/M2 polarization and related inflammatory and repair-associated genes. The primer sequences are listed in Table S2. Histological and immunohistochemical analyses, including H&E, Masson's trichrome, Sirius red, and IL-6/TNF-α staining, were performed on wound tissues collected on days 7 and 14. Collagen fiber orientation in the newly formed tissue was further analyzed using the Directionality plugin in ImageJ, and the tensile strength of healed skin was measured using a universal testing machine.

*1.11 Rabbit Ear Hypertrophic Scar Model*

A hypertrophic scar (HS) model was established on rabbit ears as previously described [6]. Briefly, male New Zealand rabbits (12–14 weeks old, 2.8–3.5 kg) were anesthetized, and the ventral surfaces of both ears were shaved and disinfected with 75% ethanol. Four full-thickness circular wounds, each 8 mm in diameter, were created on the ventral surface of each ear using sterile scalpels and scissors. The perichondrium was carefully removed while preserving the underlying cartilage. The animals were then randomly assigned to one of three groups: the HD powder group, the PBS group, or the Beifuji group (n = 6 per group). Treatments were administered for 21 days. At the end of the experiment, the animals were euthanized, and scar tissues were collected for histological evaluation, including H&E staining, Masson's trichrome staining, and Sirius red staining. Collagen fiber orientation was analyzed using the Directionality plugin in ImageJ.

*1.12 Abdominal Wound Model in Rats*

An abdominal wall incision model was established in rats as previously described [7-8]. Female Sprague–Dawley rats (8–10 weeks old, weighing 220–260 g) were used to establish the abdominal wall incision model. Prior to surgery, the abdominal area was shaved and disinfected to minimize the risk of contamination. Following anesthesia, a longitudinal skin incision approximately 2 cm in length was made on the abdomen, and the incision was closed using double-layer sutures. The animals were randomly assigned to the HD powder group, PBS group, or positive control group (n = 12 per group). Sutures were removed on postoperative day 7. Subsequently, the positive control treatment was switched from Beifuji, used during the wound healing phase, to Contractubex gel, a clinically approved topical anti-scar formulation, for ongoing scar treatment. Animals were sacrificed on postoperative days 3, 7, 14, and 21, and wound tissues were collected for histological analyses, including H&E staining, Masson's trichrome staining, Sirius red staining, immunohistochemical staining for CD31, IL-10, and TNF-α, as well as immunofluorescence staining. Collagen fiber orientation was further analyzed using the Directionality plugin in ImageJ. Additionally, the expression of inflammation- and fibrosis-related genes, including IL-10, TNF-α, TGF-β1, and TGF-β3, was quantified by RT-qPCR. Primer sequences are listed in Table S3.

*1.13 Transcriptome Sequencing*

Total RNA was extracted using TRIzol reagent according to the manufacturer's instructions. RNA integrity and concentration were assessed using an Agilent 2100 Bioanalyzer with the RNA Nano 6000 Kit. Sequencing libraries were prepared using the VAHTS Universal V6 RNA-seq Library Prep Kit. Raw sequencing data (FASTQ format) were processed with Fastp (v0.24.1) to remove adapter sequences and low-quality reads. The clean reads were aligned to the reference genome using HISAT2. Gene expression levels were quantified as fragments per kilobase of transcript per million mapped reads (FPKM), and read counts for each gene were obtained using HTSeq. Correlation analysis of RNA-seq data was performed using R (v3.2.0) to evaluate reproducibility among biological replicates. Differentially expressed genes (DEGs) were subjected to Gene Ontology (GO) and Kyoto Encyclopedia of Genes and Genomes (KEGG) pathway enrichment analyses based on a hypergeometric distribution model. R was also used to identify significantly enriched terms and to generate corresponding bar plots and bubble plots.

*1.14 HD and Cell Interaction Assays*

For fluorescence imaging experiments, RAW 264.7 and NIH-3T3 cells were seeded separately in glass-bottom culture dishes. After the cells adhered, FITC-HD was added for 1, 15, and 30 minutes. Following treatment, the cells were thoroughly washed with PBS and fixed with 4% paraformaldehyde for 15 min. Subsequently, under light-protected conditions, the cell membranes were stained with DiI (10 μM) for 15 min, followed by another PBS wash. The cell nuclei were then stained with DAPI for 10 min. Finally, PBS was added to keep the samples moist, and images were acquired using a Nikon fluorescence microscope.

*1.15 Receptor Blockade Experiments*

To evaluate the involvement of polysaccharide receptors in HD-mediated regulation of cytokine expression, receptor blockade experiments were performed. RAW 264.7 cells were pretreated for 1 h with laminin (50 μg/mL), C29 (50 μM), resatorvid (100 nM), or mannan (50 μg/mL), followed by treatment with HD (100 μg/mL) in the presence of IFN-γ (40 ng/mL) for 24 h. After treatment, the mRNA expression levels of inflammation-related cytokines were quantified by RT-qPCR.

*1.16 In Vivo CD14/TLR4 Blockade Experiment*

To investigate whether the therapeutic effects of HD are dependent on CD14/TLR4 signaling, an additional in vivo pharmacological blockade experiment was performed using the previously established deep second-degree burn model. male C57BL/6 mice (7–8 weeks old, weighing 20–24 g) were randomly assigned to four groups: PBS, HD powder, IAXO-101, and HD + IAXO-101 (n = 6 per group). To inhibit CD14/TLR4 signaling, IAXO-101 (Innaxon, UK) was freshly diluted in sterile 5% dextrose solution according to the manufacturer's instructions and administered by subcutaneous injection at a dose of 3 mg kg⁻¹ 24 h before burn induction, followed by repeated administration every 48 h throughout the experimental period. HD powder was topically applied to the burn wounds immediately after debridement according to the protocol described above.

Wound healing was monitored by digital photography throughout the experimental period. At the designated endpoint, wound tissues were harvested for total RNA extraction, and the mRNA expression levels of TNF-α, IL-6, and IL-10 were quantified by RT-qPCR. The primer sequences are listed in Table S2.

*1.17 Affinity Screening and Mass Spectrometric Identification of HD-Binding Proteins*

The affinity screening experiment was performed as previously described [10]. Briefly, HD was activated using DMTMM and covalently conjugated to the surface of carboxyl-modified latex microspheres. Membrane proteins extracted from RAW 264.7 cells were incubated with the HD-coated microspheres for 1 h. After extensive washing to remove unbound proteins, the bound proteins were eluted with SDS-PAGE loading buffer. The eluates were separated by SDS-PAGE and analyzed by LC-MS/MS. Differential enrichment analysis was then performed to identify HD-specific binding proteins and potential receptor candidates.

*1.18 Small Interfering RNA Transfection*

To suppress CD14 expression, RAW 264.7 cells were transfected with CD14-specific small interfering RNA (siRNA), while a non-targeting siRNA served as the negative control (Beijing Duona Biomedical Technology Co., Ltd.). Lipofectamine RNAiMAX was used according to the manufacturer’s instructions. Twenty-four hours after transfection, the cells were treated with HD in the presence of IFN-γ, and the expression of inflammation-related genes was quantified by RT-qPCR. The siRNA sequences are listed in Table S4.

*1.19 Molecular Docking*

The three-dimensional (3D) structure of HD was constructed using ChemDraw and converted into a suitable 3D conformation for molecular docking analysis. The crystal structure of mouse CD14 was obtained from the Protein Data Bank (PDB). The protein structure was processed using PyMOL software, and unnecessary molecules, including water molecules and non-relevant ligands, were removed before docking analysis. Molecular docking was performed using AutoDock 4 with a semi-flexible docking protocol, in which the receptor protein was treated as rigid and HD was allowed to adopt flexible conformations. The potential binding modes and interactions between HD and CD14 were predicted based on docking energy calculations. The docking conformations were further visualized and analyzed using PyMOL software to identify the potential interaction sites between HD and CD14.

*1.20 Plasmid Construction and Transfection*

The full-length mouse Cd14 cDNA was cloned into the pmCherry-N1 expression vector, and all constructs were verified by DNA sequencing. NIH-3T3 cells were transfected with the recombinant plasmid using Lipofectamine 2000 (Invitrogen). Fluorescence expression was assessed 24 h after transfection.

*1.21 Western Blot Analysis*

RAW 264.7 cells were seeded in culture plates and allowed to attach. Cells were subjected to IFN-γ stimulation and subsequently treated with different concentrations of HD, with or without CD14 functional blockade. For CD14 inhibition, RAW 264.7 cells were pretreated with an anti-mouse CD14 neutralizing antibody (clone M14-23, 10 μg/mL) prior to HD treatment. Following treatment, RAW 264.7 cells were collected for Western blot analysis of NF-κB activation by assessing phosphorylated p65 levels, and culture supernatants were collected for ELISA quantification of TNF-α, IL-6, and IL-10. For conditioned medium (CM) experiments, supernatants from treated RAW 264.7 cells were collected and centrifuged to remove cell debris. The resulting CM was used to culture HUVECs and NIH-3T3 cells. After CM incubation, HUVECs were analyzed for AKT phosphorylation, whereas NIH-3T3 cells were evaluated for Smad2 phosphorylation by Western blot and the expression of COL1A1, COL3A1, and α-SMA by RT-qPCR.

Following treatment, RAW 264.7 cells, HUVECs, and NIH-3T3 cells were lysed in RIPA buffer supplemented with protease and phosphatase inhibitors. Total protein concentrations were determined using a BCA protein assay kit. Equal amounts of proteins were separated by sodium dodecyl sulfate–polyacrylamide gel electrophoresis and transferred onto polyvinylidene fluoride membranes. After blocking, membranes were incubated with specific primary antibodies overnight at 4 °C, followed by incubation with horseradish peroxidase-conjugated secondary antibodies. Protein bands were visualized using enhanced chemiluminescence (ECL), and band intensities were quantified using ImageJ software (National Institutes of Health). Phosphorylated protein levels were normalized to their corresponding total protein levels, whereas total protein expression was normalized to β-actin.

*1.22 Evaluation of the Cell Regulatory Functions of HD Powder*

1.22.1 In Vitro Antioxidant Activity

The intracellular antioxidant activity of HD was evaluated using a ROS assay kit, following the manufacturer's instructions. RAW 264.7 cells were seeded at a density of 2 × 10⁵ cells per well in 6-well plates. After 12 h of incubation, the cells were loaded with DCFH-DA diluted in serum-free medium for 4 h. The culture medium was then replaced with fresh medium in the negative control group. Cells in the positive control group were treated with H_2_O_2_ to induce oxidative stress, while cells in the HD treatment groups were exposed to H_2_O_2_ along with either low or high doses of HD. After incubation at 37 °C for 30 min, fluorescence images were captured using an inverted fluorescence microscope (Ti2, Nikon), and DCF fluorescence was detected at an excitation wavelength of 488 nm.

1.22.2 Cell Scratch Assay

NIH-3T3 cells were seeded at a density of 5 × 10⁵ cells per well in 24-well plates and cultured for 24 h to form a confluent monolayer. A sterile 10 μL pipette tip was used to create a vertical scratch in the center of each well. Detached cells were gently removed by washing with PBS. RAW 264.7 cells were stimulated with IFN-γ (40 ng/mL) in the presence or absence of HD (10 or 100 μg/mL) for 24 h. The culture supernatants were collected, centrifuged to remove cell debris, and used as CM. NIH-3T3 cells were subsequently cultured with the indicated CM. CM collected from untreated RAW 264.7 cells and IFN-γ-stimulated RAW 264.7 cells served as the control and model groups, respectively. Images of the scratch area were captured at 0, 12, and 24 h using an inverted fluorescence microscope. The wound closure area was quantified using ImageJ to evaluate cell migration.

1.22.3 Cell Tube Formation Assay

The effect of HD on angiogenesis was evaluated using a tube formation assay, as previously described[12]. RAW 264.7 cells were pretreated with or without an anti-CD14 neutralizing antibody for 1 h, followed by stimulation with IFN-γ (40 ng/mL) in the presence or absence of HD (10 or 100 μg/mL) for 24 h. Culture supernatants were collected, centrifuged to remove cell debris, and used as CM. HUVECs were cultured with the indicated CM for 24 h, after which the cells were harvested and seeded onto Matrigel-coated 96-well plates at a density of 1.5 × 10⁴ cells per well. Untreated cells served as the control group. After incubation for 6 h at 37 °C in a humidified atmosphere containing 5% CO₂, tube formation was imaged using an inverted fluorescence microscope. The number of tubular structures was quantified using ImageJ software.

2. **Supplementary Experimental Results**

**Fig. S1** HD exhibits good cytocompatibility in RAW 264.7 macrophages at the tested concentrations. Cell viability of raw 246.7 cells treated with different concentrations of HD samples (n=3).

**Fig. S2** HD exhibits good hemocompatibility. Hemolysis assay of HD treatment (n=3).

**Fig. S3** HD undergoes progressive degradation and clearance with transient systemic distribution in vivo. In vivo fluorescence tracking of FITC-labeled HD at the implantation site, in plasma, and in major organs at predetermined time points (n = 3).

**Fig. S4** HD undergoes progressive degradation accompanied by cellular infiltration and tissue integration. Fluorescence microscopy showing the temporal changes in FITC-labeled HD degradation and host cell infiltration at the implantation site.

**Fig. S5** HD promotes collagen remodeling and reduces excessive collagen deposition in burn wounds. Quantitative analysis of Sirius Red staining in burn wound tissues on day 14 (n = 3). Statistical significance was analyzed using one-way ANOVA (***p* < 0.01, ****p* < 0.001). Error bars represent standard deviation.

**Fig. S6** HD promotes orderly collagen fiber alignment during wound healing. Distribution of collagen fiber orientation in burn wound tissues.

**Fig. S7** HD reduces excessive collagen deposition in the rabbit ear hypertrophic scar model. Quantitative analysis of Sirius Red staining in rabbit ear scar tissues on day 21 (n = 3). Statistical significance was analyzed using one-way ANOVA (**p* < 0.05, ***p* < 0.01, ****p* < 0.001), ns indicates no significant difference. Error bars represent standard deviation.

**Fig. S8** HD restores organized collagen architecture in hypertrophic scar tissues. Distribution of collagen fiber orientation in the rabbit ear model on day 21.

**Fig. S9** HD modulates inflammatory and fibrotic gene expression in wounds tissues. Relative mRNA expression levels of IL-10, TNF-α, TGF-β1, and TGF-β3 determined by RT-qPCR on day 21 (n = 3). Statistical significance was analyzed using one-way ANOVA (**p* < 0.05, ***p* < 0.01, ****p* < 0.001), ns indicates no significant difference. Error bars represent standard deviation.

**Fig. S10** HD improves tissue regeneration and collagen remodeling. Representative H&E and Masson's trichrome staining on day 14.

**Fig. S11** HD promotes collagen remodeling in wound tissues. Quantitative analysis of Sirius Red staining in wound tissues on day 14 (n = 3). Statistical significance was analyzed using one-way ANOVA (***p* < 0.01, ****p* < 0.001), ns indicates no significant difference. Error bars represent standard deviation.

**Fig. S12** HD improves collagen fiber organization and extracellular matrix remodeling during wound healing. Distribution of collagen fiber orientation in wound tissues.

**Fig. S13** HD modulates the expression of wound healing-associated proteins in wound tissues. Quantitative analysis of immunofluorescence staining on day 21 (n = 3). Statistical significance was analyzed using one-way ANOVA (**p* < 0.05, ***p* < 0.01, ****p* < 0.001), ns indicates no significant difference. Error bars represent standard deviation.

**Fig. S14** HD promotes peripheral nerve regeneration in wound tissues. Representative β3-tubulin immunofluorescence staining in wound tissues on day 21.

**Fig. S15** HD promotes tissue regeneration in wound tissues. Quantitative analysis of immunofluorescence staining on day 21 (n = 3). Statistical significance was analyzed using one-way ANOVA (**p* < 0.05, ****p* < 0.001), ns indicates no significant difference. Error bars represent standard deviation.

**Fig. S16** FITC-labeled HD rapidly interacts with RAW 264.7 macrophages. Confocal fluorescence images showing the interaction of FITC-labeled HD (green) with RAW 264.7 macrophages at 1 and 15 min.

**Fig. S17** Pharmacological inhibition of the CD14/TLR4 axis attenuates the therapeutic efficacy of HD during burn wound healing. (a) Treatment protocol of the deep second-degree burn model. (b) Representative photographs of wounds during the healing process. (c) Quantification of wound closure (n = 3). Relative mRNA expression levels of (d) IL-6 and (e) TNF-α in wound tissues determined by RT-qPCR (n = 3). Statistical significance was analyzed using one-way ANOVA (**p* < 0.05, ***p* < 0.01, ****p* < 0.001). Error bars represent standard deviation.

**Fig. S18** Western blot images showing p65 and p-p65 in RAW 264.7 cells, Smad2 and p-Smad2 in NIH-3T3 cells, and AKT and p-AKT in HUVECs (n=3).

 **Fig. S19** Effects of CD14 neutralization on the immunomodulatory activity of HD in IFN-γ-stimulated RAW264.7 macrophages and the subsequent paracrine regulation of fibroblast fibrotic gene expression (n = 3). RAW264.7 macrophages were pretreated with the anti-CD14 neutralizing antibody M14-23 prior to HD stimulation. The concentrations of IL-6, TNF-α, and IL-10 in the culture supernatants were determined by ELISA. Conditioned media from macrophages, with or without CD14 blockade, were subsequently transferred to fibroblasts, and the mRNA expression levels of COL1A1, COL3A1, and α-SMA were analyzed by qRT-PCR. ** *p* < 0.01, *** *p* < 0.001 vs. the CK group. # *p* < 0.05, ## *p* < 0.01, ### *p* < 0.001 vs. the IFN-γ group. && *p* < 0.01, &&& *p* < 0.001 vs. the HD (100 μg/mL) group. Statistical significance was analyzed using one-way ANOVA and an unpaired Student's t-test. Error bars represent standard deviation.

**Fig. S20** Quantitative analysis of HUVEC tube formation showing enhanced angiogenic capacity in HD-conditioned microenvironment, reversed by CD14 neutralization. Statistical significance was analyzed using one-way ANOVA (***p* < 0.01), ns indicates no significant difference. Error bars represent standard deviation.

**Fig. S21** Dynamic regulation of macrophage polarization and inflammatory cytokine expression during HD-mediated burn wound healing. Representative immunofluorescence images of iNOS (yellow), Arg-1 (green), and F4/80 (red) staining in burn wound tissues at days 3, 7, and 14 after treatment. Quantification of iNOS⁺/F4/80⁺ macrophages and Arg-1⁺/F4/80⁺ macrophages. Relative mRNA expression levels of IL-6 and TNF-α in wound tissues were determined by qRT-PCR (n = 3).

**Table S2.** The sequences of primers used in RT-qPCR

| **Genes** | **Forward primers (5’-3’)** | **Reverse primers (5’-3’)** |
| --- | --- | --- |
| GAPDH (Mouse) | CCTCGTCCCGTAGACAAAATG | TGAGGTCAATGAAGGGGTCGT |
| IL-10 (Mouse) | AATAAGCTCCAAGACCAAGGTGT | CATCATGTATGCTTCTATGCAGTTG |
| TGF-β1 (Mouse) | CATTGCTGTCCCGTGCAGA | AGGTAACGCCAGGAATTGTTGCTA |
| TGF-β3 (Mouse) | CCGGATGAGCACATAGCCAA | TCTCTCTCTCCAACAGCCACTCG |
| COL1A1 (Mouse) | GACAGGCGAACAAGGTGACAGAG | CAGGAGAACCAGGAGAACCAGGAG |
| COL3A1 (Mouse) | ACGAGGTGACAAAGGTGAAACTGG | AGAACCTGGAGGACCTGGATTGC |
| α-SMA (mouse) | CCAGCACCATGAAGATCAAG | TCCACATCTGCTGGAAGGTA |
| CD14 (Mouse) | AACTCGCTCAATCTGTCTTTCAC | GCTCATCTGGGCTAGGGTTC |
| IL-1β (Mouse) | GAGCTTCAGGCAGGCAGTATC | GTATAGATTCTTTCCTTTGAGGC |
| TNF-α (Mouse) | GACAGACTACACGACATTGAG | ATGGTCACAGTTGGGAGCATC |
| IL-6 (Mouse) | CTCTCTGCAAGAGACTTCCA | CTCTCCGGACTTGTGAAGTA |
| CXCL1 (Mouse) | CTGGGATTCACCTCAAGAACATC | CAGGGTCAAGGCAAGCCTC |

**Table S3.** The sequences of primers used in RT-qPCR

| **Genes** | **Forward primers (5’-3’)** | **Reverse primers (5’-3’)** |
| --- | --- | --- |
| GAPDH(Rat) | GCATCTTCTTGTGCAGTGCC | GACTGTGCCGTTGAACTTGC |
| TNF-α（Rat） | CTCAGAGCCCCCAATCTGTG | ACTCAGGCATCGACATTCC |
| IL-10 (Rat) | AATAAGCTCCAAGACCAAGGTGT | CATCATGTATGCTTCTATGCAGTTG |
| TGF-β1 (Rat) | GGCGGTGCTCGCTTTGTA | TCCCGAATGTCTGACGTATTGA |
| TGF-β3 (Rat) | CCTGGCCCTGCTGAACTTG | TTGATGTGGCCGAAGTCCAAC |

**Table S4.** Sequences of siRNA

| **Targets** | **Sense (5′3′)** | **Antisense (5′3′)** |
| --- | --- | --- |
| mCD14 siRNA-1 | GCCUGGAAUACCUUCUAAATT | UUUAGAAGGUAUUCCAGGCTT |
| mCD14 siRNA-2 | GGGCAGUUCACUGAUAUUATT | UAAUAUCAGUGAACUGCCCTT |
| mCD14 siRNA-3 | CAGGAACUGACUCUUGAAATT | UUUCAAGAGUCAGUUCCUGTT |
| siRNA-NC | UUCUCCGAACGUGUCACGUTT | ACGUGACACGUUCGGAGAATT |

**References**

[1]	C. Kong, S. Chen, X. Wang, et al., “Hemoadhican, a Tissue Adhesion Hemostatic Material Independent of Blood Coagulation,” *Adv. Healthc. Mater.* **2023**, *12* (24), e2300705, <https://doi.org/10.1002/adhm.202300705>.

[2]	F. Cheng, C. Liu, X. Wei, et al., “Preparation and Characterization of 2,2,6,6-Tetramethylpiperidine-1-oxyl (TEMPO)-Oxidized Cellulose Nanocrystal/Alginate Biodegradable Composite Dressing for Hemostasis Applications,” *ACS Sustainable Chem. Eng.* **2017**, *5* (5), 3819, <https://doi.org/10.1021/acssuschemeng.6b02849>.

[3]	Y. Liu, R. Zheng, H. Ren, et al., “A self-fixing xanthan gum hydrogel membrane with ROS scavenging capability for the prevention of postoperative abdominal adhesion,” *Int. J. Biol. Macromol.* **2025**, *289*, 138676, [https://doi.org/10.1016/j.ijbiomac.2024.138676](https://doi.org/https://doi.org/10.1016/j.ijbiomac.2024.138676).

[4]	L. Chai, J. Huang, M. Wang, et al., “Injectable deferoxamine-loaded microsphere hydrogels for inhibition of ferroptosis and promotion of third-degree burn wound healing,” *Mater Today Bio* **2025**, *32*, 101806, <https://doi.org/10.1016/j.mtbio.2025.101806>.

[5]	Z. Chen, Z. Zhou, X. Zhang, et al., “A carboxymethyl chitosan and dextran hydrogel with slow and rapid photothermal conversion for sequential promoting burn wound healing and inhibiting scar proliferation,” *Carbohydr. Polym.* **2025**, *350*, 123045, <https://doi.org/10.1016/j.carbpol.2024.123045>.

[6]	R. Fang, S. Chen, Q. Liu, et al., “An AIE-Active Polyvinyl Alcohol/Berberine/Hemoadhican Smart Hydrogel for Scarless Wound Healing with Visual pH Monitoring,” *Mater. Today Bio* **2026**, 103224, [https://doi.org/10.1016/j.mtbio.2026.103224](https://doi.org/https://doi.org/10.1016/j.mtbio.2026.103224).

[7]	D. Bowers, D. McKenzie, D. Dutta, et al., “Growth hormone treatment after cesarean delivery in rats increases the strength of the uterine scar,” *Am. J. Obstet. Gyneco.l* **2001**, *185* (3), 614, <https://doi.org/10.1067/mob.2001.117185>.

[8]	Z. Li, L. Yang, Q. Jin, et al., “An anti-inflammatory and anti-fibrotic Janus hydrogel for preventing postoperative peritoneal adhesion,” *Mater. Today Bio* **2025**, *31*, 101637, [https://doi.org/10.1016/j.mtbio.2025.101637](https://doi.org/https://doi.org/10.1016/j.mtbio.2025.101637).

[9]	D. Yu-Hao, C. Chun, F. Xiong, et al., “Study on the pharmacokinetics of mulberry fruit polysaccharides through fluorescence labeling,” *Int. J. Biol. Macromol.* **2021**, *186*, 462, [https://doi.org/10.1016/j.ijbiomac.2021.07.075](https://doi.org/https://doi.org/10.1016/j.ijbiomac.2021.07.075).

[10]	H. An, X. Tian, Y. Huang, et al., “Identification of the mouse Kupffer cell receptors recognizing pneumococcal capsules by affinity screening,” *STAR Protoc.* **2023**, *4* (1), 102065, [https://doi.org/10.1016/j.xpro.2023.102065](https://doi.org/https://doi.org/10.1016/j.xpro.2023.102065).

[11]	Z. Li, K. M. Bratlie, “Fibroblasts treated with macrophage conditioned medium results in phenotypic shifts and changes in collagen organization,” *Mater. Sci. Eng. C* **2021**, *122*, 111915, [https://doi.org/10.1016/j.msec.2021.111915](https://doi.org/https://doi.org/10.1016/j.msec.2021.111915).

[12]	H. Xue, C. Zhang, D. Lin, et al., “Isoliquiritigenin micellar microneedle for pH monitoring and diabetic wound healing,” *Mater. Today Bio* **2025**, *35*, 102356, [https://doi.org/10.1016/j.mtbio.2025.102356](https://doi.org/https://doi.org/10.1016/j.mtbio.2025.102356).
